# Hemi-Synthesis of Chiral Imine, Benzimidazole and Benzodiazepines from Essential Oil of *Ammodaucus leucotrichus* subsp. *leucotrichus*

**DOI:** 10.3390/molecules24050975

**Published:** 2019-03-10

**Authors:** Farid Chebrouk, Khodir Madani, Brahim Cherfaoui, Leila Boukenna, Mónica Válega, Ricardo F. Mendes, Filipe A. A. Paz, Khaldoun Bachari, Oualid Talhi, Artur M. S. Silva

**Affiliations:** 1 Laboratory of Biomathematics, Biochemistry, Biophysics and Scientometrics (L3BS), Faculty of Nature and Life Sciences (FSNV), University of Bejaia, 06000 Bejaia, Algeria; chebroukfarid@yahoo.fr; 2 Research Center Scientific and Technical in Analyzes Physico-Chimiques CRAPC, BP384, Bou-Ismail, 42004 Tipaza, Algeria; cherfaoui.br@gmail.com (B.C.); lboukenna15@gmail.com (L.B.); bachari2000@yahoo.fr (K.B.); 3 QOPNA and LAQV-REQUIMTE, Department of Chemistry, University of Aveiro, 3810-193 Aveiro, Portugal; mvalega@ua.pt; 4 CICECO—Aveiro Institute of Materials, Department of Chemistry, University of Aveiro, 3810-193 Aveiro, Portugal; rfmendes@ua.pt (R.F.M.); filipe.paz@ua.pt (F.A.A.P.); 5 Laboratory of Applied Organic Chemistry, Faculty of Chemistry, University of Science and Technology Houari Boumediene, BP 32, Alia Bab-Ezzouar, 16111 Algiers, Algeria

**Keywords:** (*S*)-(−)-perillaldehyde, *Ammodaucus leucotrichus*, amines, hemi-synthesis, essential oil, 2D NMR, single-crystal X-ray diffraction, chiral-HPLC

## Abstract

The hemi-synthesis of chiral imine, benzimidazole and benzodiazepine structures is reported by the condensation of (*S*)-(−)-perillaldehyde, the major phytochemical of *Ammodaucus leucotrichus* subsp. *leucotrichus* essential oil, with different amine derivatives of 2,3-diaminomaleonitrile, *o*-phenylenediamine and 3-[(2-aminoaryl)amino]dimedone. The reaction proceeds in situ at ambient temperature without prior isolation of the natural (*S*)-(−)-perillaldehyde. Final products precipitate in the ethanolic reaction medium. 2D NMR and single-crystal X-ray diffraction studies were used to unequivocally characterize the structures in solution and in the solid state, respectively. Chiral HPLC analysis confirms the formation of unique enantiomers and diastereomeric mixtures.

## 1. Introduction

Over the past decades, there has been a growing transition in drugs from natural materials, natural products, or their simple derivatives to more potent natural-mimicking synthetic prototypes [1]. Essential oils’ components still play a major role in this combinatorial chemistry and are considered promising sources of stereospecific structures. They can indeed serve as substrates in hemi-synthesis, leading to new active molecules. For instance, (+)-carotol, the major constituent of carrot seed essential oil, was used as a starting material in the hemi-synthesis of ten cytotoxic hydroindene-derived chiral synthons [2]. A novel series of essential oils oriented chiral esters with high insecticidal activity have been synthesized based on the scaffold of the natural pyrethrin [3]. *Cymbopogon schoenanthus* essential oil is mainly composed of piperitone (68.2%), which was used for in situ preparation of three anti-parasitic carbasones [4].

*Ammodaucus leucotrichus Cosson & Durieu* subsp. *leucotrichus* belongs to the *Apiaceae* (*Umbelliferae*) plant family. It is an endemic species growing in the Saharan and sub-Saharan countries of North Africa, extending up to Egypt and tropical Africa [5]. Generally, it grows in wadis with granite boulders (temporary water streams) [6]. This plant plays an important role in traditional medicine in North African countries. It is used as a remedy for cardiac diseases [7], rheumatism, asthma and stomach diseases [8,9]. The major components in the essential oil of *A. leucotrichus* from Morocco were reported to be perillaldehyde (63.6%) and limonene (26.8%) [5]. A similar phytochemical profile is reported for the essential oil of *A. leucotrichus* growing wild at different areas in the Southern Algerian Sahara, characterized by perillaldehyde (37.5–84.4%) and limonene (7.0%–29.2%) [10,11,12]. (*S*)-(−)-Perillaldehyde laevorotatory form is found most abundantly in essential oils of other plants such as the woody shrub *Conyza newii* [13]. This aldehyde is extensively used as a food additive for flavouring and especially as an ingredient in perfumes [14], but it also exhibits antioxidant, antidepressant and other biological activities [15].

In general, aldehydes are most commonly used in the synthesis of a large range of interesting molecules, such as imines and benzodiazepines. Many preparations of various imines by the condensation of different aldehydes with aliphatic or aromatic amines are reported in the literature [16,17]. This kind of molecule is known to possess significant biological properties, such as antitumor, insecticidal, antibacterial, anti-tuberculosis, antimicrobial and anticonvulsant activities [18]. Benzodiazepines represent a well-known class of therapeutics displaying anticonvulsant, anti-inflammatory, analgesic and anti-depressive effects [19]. A literature survey reveals that a fair amount of work has been published in the stereospecific synthesis of benzodiazepine derivatives by the condensation of aldehydes and several heterocyclic compounds [20,21]. In particular, a simple and fast method has recently been described by us for the elaboration of novel benzodiazepine structures [22].

Following this last method, we propose herein the hemi-synthesis of novel chiral imine, benzimidazole and benzodiazepine structures from the essential oil of *A. leucotrichus* by condensation of its major constituent (*S*)-(−)-perillaldehyde (81.0%) with different amine substrates, such as 2,3-diaminomaleonitrile, *o*-phenylenediamine and 3-[(2-aminoaryl)amino]dimedone. The GC-MS analysis of our *A. leucotrichus* essential oil allowed the identification of perillaldehyde (81.0%) and limonene (12.4%) as major constituents (see Supplementary Materials). The combination of amines with the enantiopure perillaldehyde may lead to functional synergy and render the final chiral molecules with improved biological properties, since it is well-known that the biological effect is intrinsically related to one of the enantiomers. In addition, the toxicological, pharmacokinetic behaviour and metabolism of the enantiomers may also be different [23].

## 2. Results and Discussion

### 2.1. Chemistry and Mechanism

**Scheme 1:**
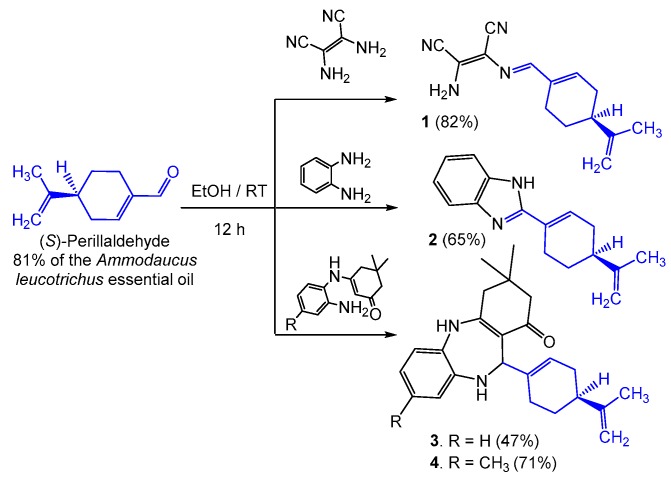
In situ hemi-synthesis of imine **1**, benzimidazole **2** and benzodiazepines **3**–**4** from the main constituent of the *Ammodaucus leucotrichus* essential oil.

Perillaldehyde, the major constituent of the *A. leucotrichus* essential oil, reacts in situ with the aforementioned amines without the need for prior purification or isolation from the oil matrix. The condensation reaction proceeds under similar operating conditions as previously published [22], by employing ethanol as a solvent at ambient temperature and under catalyst-free conditions over a period of 12 h. The reaction is easily worked up by filtration of the formed solid showing a high purity of products **1**–**4**, isolated in moderate-to-good yields (47%–82%) after washing with ethanol, ultimately avoiding any further chromatographic purifications (Scheme 1).

**Scheme 2:**
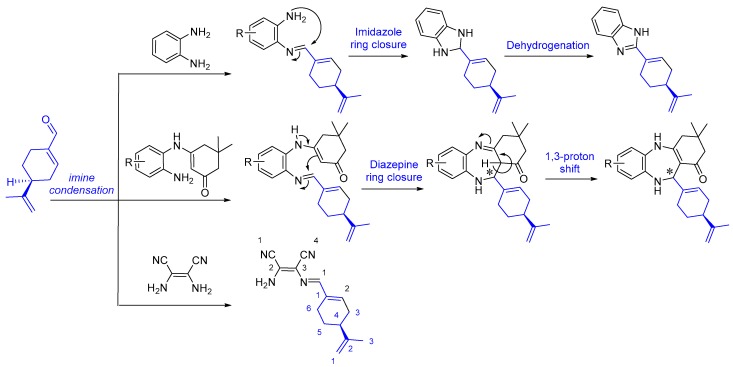
Mechanistic pathway for the hemi-synthesis of imine **1**, benzimidazole **2** and benzodiazepine **3**–**4** structures form essential oil of *A. leucotrichus* in situ.

The final products **1**–**4** are chiral because the condensation reactions are carried out over the aldehyde group, ultimately maintaining the configuration of the asymmetric centre of (*S*)-perillaldehyde. This has direct implications in the performed crystallographic studies as detailed below. The reaction mechanisms follow the classical imine condensation to afford compound **1**, 2-amino-3-{[(*E*)-[(*S*)-4-(prop-1-en-2-yl)cyclohex-1-en-1-yl)methylene]amino}maleonitrile, obtained from the reaction of the main constituent of the *A. leucotrichus* essential oil with 2,3-diaminomaleonitrile (Scheme 2). In a similar way, *o*-phenylenediamine condenses on perillaldehyde to afford the corresponding imine as an intermediate. The latter undergoes a second amine attack, giving rise to an imidazole ring closure after spontaneous dehydrogenation at ambient temperature, therefore generating (*S*)-2-[4-(prop-1-en-2-yl)cyclohex-1-en-1-yl]-1*H*-benzo[*d*]imidazole **2** via a tandem process (Scheme 2). In common reaction cases, the imidazole ring closure occurs at higher temperatures (heating at reflux) [24]. However, our experimental protocols were strategically oriented to perform hemi-synthetic transformations at room temperature, which allows a rapid precipitation/crystallization of the final chiral product upon formation. Under these relatively low temperature conditions, compound **1** could not cyclize to imidazole, while the benzimidazole ring closure in compound **2** was possible due to chemical stabilization of the mentioned heterocyclic system. The last experiment was performed using 3-[(2-aminoaryl)amino]dimedone which, according to our previous study [22], originates 3,3-dimethyl-11-[(*S*)-4-(prop-1-en-2-yl)cyclohex-1-en-1-yl]-2,3,4,5,10,11-hexahydro-1*H*-dibenzo[*b,e*][1,4]diazepin-1-ones **3** and **4** via imine condensation and diazepine ring closure that creates a new asymmetric centre over the diazepine ring (Scheme 2).

### 2.2. Chiral HPLC and Stereochemistry

**Figure 1:**
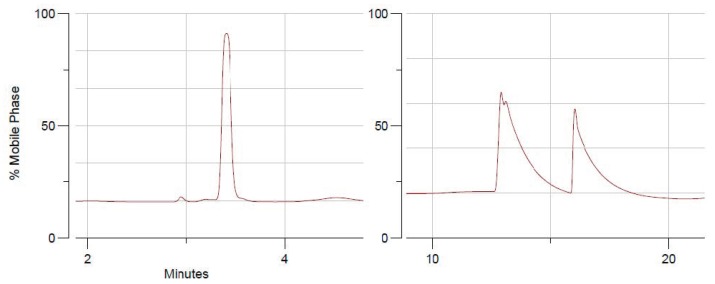
Chiral-HPLC separation of compound **1** (pure enantiomer—**left**) and compound **3** (diasteromeric mixture—**right**).

(*S*)-(−)-Perillaldehyde is found in essential oils in an optically active laevorotatory form due to the presence of an asymmetric carbon [13]. This carbon was chemically resistant to our amine nucleophilic attacks on perillaldehyde where chemical alterations were mainly brought to the aldehyde function to produce compounds **1**–**4** (including imine, imidazole and 1,4-diazepines) and maintaining the asymmetric carbon unchanged. This fact was further studied, however, by chiral-HPLC analysis performed on imine **1**, imidazole **2** and 1,4-benzodiazepine **3**, where the presence of a sole chromatographic peak indicated the existence of one enantiomer as noted in the case of compounds **1** and **2** (Figure 1, see Supplementary Materials). Structurally, 1,4-benzodiazepines **3** and **4** are composed of the asymmetric perillaldehyde moiety attached to the new connecting asymmetric carbon which belongs to the diazepine ring. Stereochemical and mechanistic considerations suggest the presence of a 1:1 ratio of two diastereomers (11-*R*, 4′-*S*- and 11-*S*, 4′-*S*-isomers) (Scheme 2). This was proved with the help of chiral-HPLC, showing the presence of two chromatographic peaks with 1:1 integrating areas, ultimately supporting the formation of a diastereomeric mixture of 1,4-benzodiazepine **3** (see Supplementary Materials). According to chiral chromatographic profiles of **1**–**3**, our hemi-synthetic process was efficient without any side products being formed, showing high purity of the unique enantiomeric forms of compounds **1** and **2**, along with diastereomeric mixtures of 1,4-benzodiazepines **3** and **4**.

### 2.3. Nuclear Magnetic Resonance Spectroscopy

**Figure 2:**
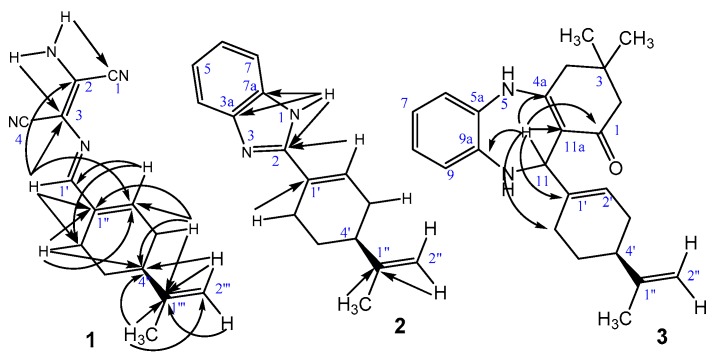
HMBC correlations of compound **1**–**3**.

The structures of **1**–**4** were characterized by 1D and 2D NMR spectroscopy (see Supplementary Materials). The ^1^H-NMR spectrum of compounds **1**–**4** suggested the presence of the asymmetric perillalkyl unit, which is mainly characterized by aliphatic protons of methylene groups appearing as multiplets between δ_H_ 1.0 and 3.0 ppm with their positions confirmed based on DEPT-135, HSQC and HMBC experiments (Figure 2). Vinylic protons of the perillalkyl moiety were attributed around 4.40–5.00 ppm (for the extra-cyclic protons) and 5.00–6.70 ppm (for the intra-cyclic ones). Using DEPT-135 and HSQC experiment, all the protonated carbons were assigned, especially those from δ_C_ 20 to 40 ppm due to aliphatic methyl and methylene groups of perillalkyl radical (see Supplementary Materials).

The structures of **1**–**4** were further confirmed based on the HMBC experiments. The main HMBC connectivities observed over the perillalkyl unit are depicted in Figure 2. Aromatic and vinylic quaternary carbons were localized via HMBC correlations with their neighbouring protons, especially in the case of 1,4-benzodiazepines **3** and **4**, where the proton H-11 was shown to establish four different connectivities with the surrounding quaternary carbons, along with the carbonyl C-1 (Figure 2). A deep examination of both ^1^H and ^13^C-NMR spectra of 1,4-benzodiazepines **3** and **4** revealed the presence of duplicated signals with a 1:1 ratio according to proton integration, which is clear evidence of the presence of diastereomeric mixtures (see Supplementary Materials).

### 2.4. Single-Crystal X-ray Diffraction

**Figure 3:**
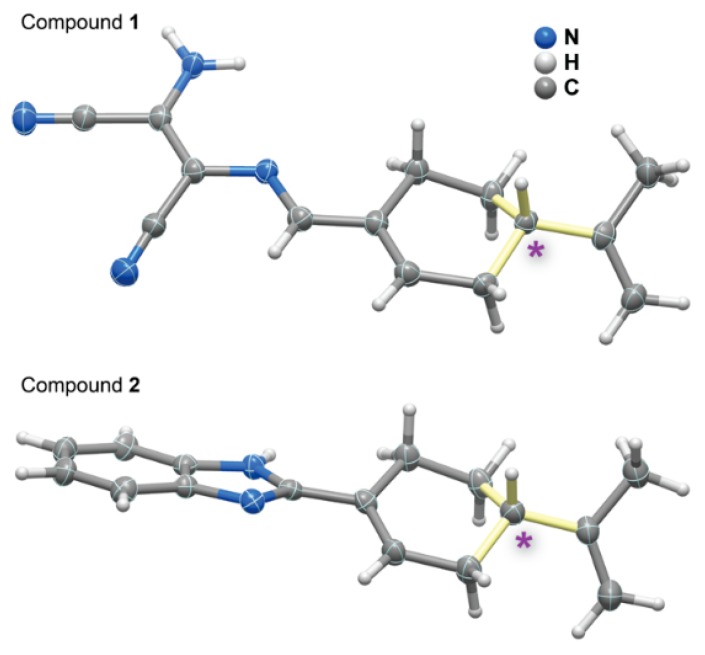
Schematic representation of molecular units present in the crystal structures of compounds **1** and **2** (note: the second identical molecule present in the asymmetric unit of **2** was omitted for clarity). Asymmetric carbon centres are depicted by an asterisk and the bonds in light yellow. Non-hydrogen atoms are represented as thermal ellipsoids drawn at the 50% probability level and hydrogen atoms as small spheres with arbitrary radii.

**Figure 4:**
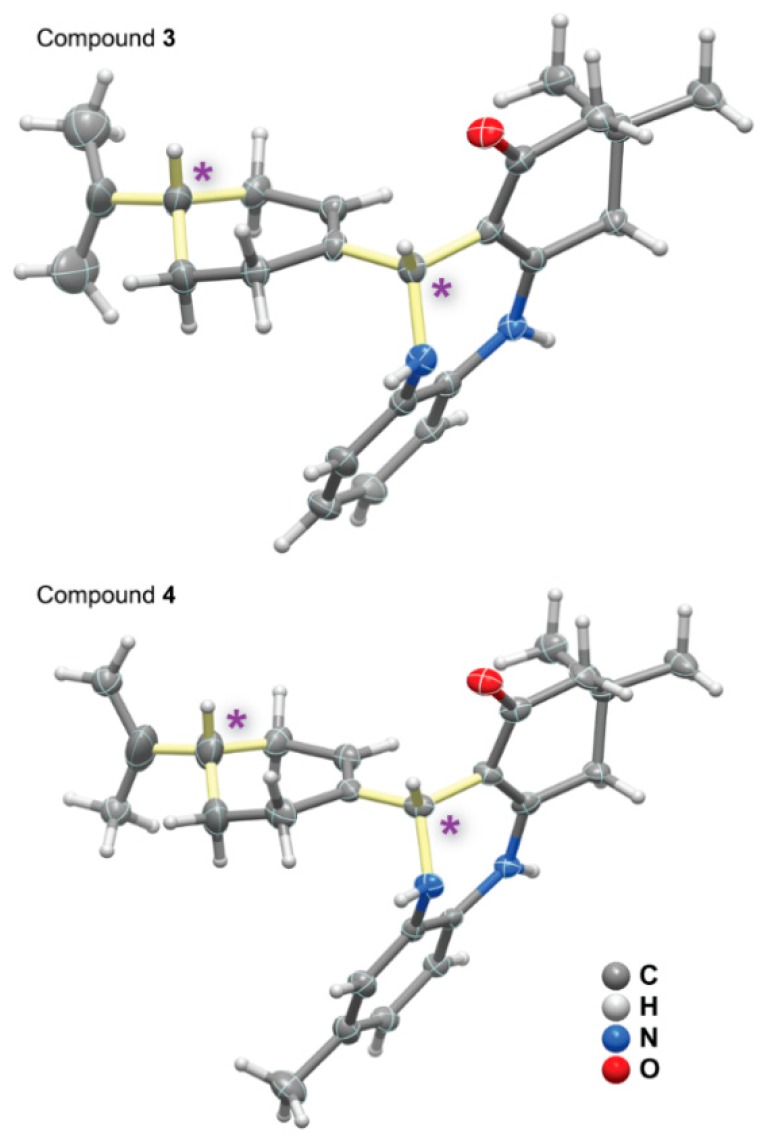
Schematic representation of the (*S*,*S*)-diastereomers molecular units present in the crystal structures of compounds **3** and **4** (note: the (*S*,*R*)-diastereomer molecules present in the asymmetric units were omitted for clarity). Asymmetric carbon centres are depicted by an asterisk and the bonds are in light yellow. Non-hydrogen atoms are represented as thermal ellipsoids drawn at the 50% probability level and hydrogen atoms as small spheres with arbitrary radii.

Single-crystal X-ray diffraction studies were further used to determine the spatial arrangement of the new compounds **1**–**4** (Figure 3 and Figure 4). Crystals of compounds **1**–**4** were directly obtained from the reaction batches after filtration and washing with ethanol. This is noteworthy because use of the natural (*S*)-(−)-perillaldehyde as a starting chiral reagent means that all the resulting structures crystallized in chiral and non-centrosymmetric space groups. The absence of strongly diffracting elements in all structures means that the absolute configurations of asymmetric carbons could not be determined from the X-ray studies, but they were nevertheless unveiled from the chiral HPLC studies performed.

Compounds **1** and **2** only have a single asymmetric carbon centre belonging to the parent perillalkyl unit, exhibiting the (*S*)-configuration (Figure 3). While **1** crystallized in a non-centrosymmetric monoclinic space group *P*2_1_ with the asymmetric unit having only one molecule, **2** crystallized in the non-centrosymmetric orthorhombic space group *P*2_1_2_1_2_1_, with two molecules composing the asymmetric unit. These two molecules had the same configuration for the asymmetric carbon centre with the inequivalence arising from simple rotations of the various molecular units composing the molecule. The presence of donor and acceptor units capable of engaging in strong hydrogen bonds was well evidenced in the crystal packing of both **1** and **2**. In **1**, the two nitrile groups and the amine moiety were engaged in strong intermolecular N–H···N hydrogen bonds, forming a **R^3^_3_**(14) graph set (*d*_N···N_ = 3.012(3)–3.028(3) Å and <(NHN) = 147–168°), ultimately dictating the way the molecules closely pack in the solid state. In **2**, there was a single N–H···N interaction connecting the two crystallographically independent molecular units, leading to the formation of a **C^2^_2_**(8) graph set motif (*d*_N···N_ = 2.853(4) –2.854(4) Å and <(NHN) = 163°) [25].

Compounds **3** and **4** both crystallized in the non-centrosymmetric monoclinic *I*2 space group, with the asymmetric units being composed of a pair of (*S,S*)- and (*R,S*)-diastereomers (in Figure 4 only the (*S,S*)-diastereomers are depicted for the two compounds). According to our previous mechanistic descriptions, the diazepine cyclisation leads to the formation of an extra asymmetric carbon (C-11), with the one from perillaldehyde (C-4′) always maintaining its original (*S*)-configuration. We further note that chiral HPLC clearly showed the presence of a mixture of two diastereomers, which agrees with the crystallographic studies performed. The configuration of all the stereocentres was determined to be (11-*R*,4′-*S*) and (11-*S*,4′-*S*). These results are also in agreement with the aforementioned 2D-NMR investigations.

## 3. Materials and Methods

### 3.1. General Remarks

Melting points were measured with a Büchi Melting Point B-540 apparatus (BÜCHI Labortechnik AG, Flawil, Switzerland). NMR spectra were recorded on a Bruker AVANCE 500 spectrometer (Bruker, Wissembourg, France, 400 MHz for ^1^H and 100 MHz for ^13^C), in DMSO-d_6_ as solvent. Chemical shifts (δ) were reported in ppm and coupling constants (*J*) in Hz and the internal standard was TMS. Unequivocal ^13^C assignments were made with the aid of 2D gHSQC and gHMBC experiments (delays for one-bond and long-range *J*_C/H_ couplings were optimized for 145 and 7 Hz, respectively). High-resolution mass spectra (ESI^+^-HRMS) were measured with a micrOTOF-Q 98 spectrometer (Bruker Daltonics, Hamburg, Germany). GC-MS analysis of *A. leucotrichus* subsp. *leucotrichus* essential oil was performed on a Hewlett-Packard computerized system comprising a 6890 gas chromatograph coupled with a 5973A mass spectrometer (Agilent Technologies, Santa Clara, CA, United States). All chemicals and solvents were purchased from commercial sources and were used as received. Chiral-HPLC separation of compounds **1**–**3** was performed on chiral stationary phase at 25 °C using a CHIRALPAK^®^ IA (Chiral Technologies Europe, Illkirch, France, amylose-tris(3,5-dimethylphenylcarbamate) immobilized on 5 μm silica-gel, 250 mm × 4.6 mm ID). The mobile phase used was hexane/acetone (isocratic mode, 50:50 (*v*/*v*)) at a flow rate of 1.0 mL/min. The UV detector was set at 220 nm (Figure S21 in the Supplementary Information). An injection of 20 μL of 1.0 g/L concentrated samples of dissolved compounds **1**–**3** was used in the mobile phase.

### 3.2. Plant Material and Extraction Procedure

The aerial parts of *A. leucotrichus* were collected from Ghardaia (Septentrional Algerian Sahara) in April 2017. They were identified by the botanists of the National Agronomic Institute in El-Harrach Algeria. Air-dried fruits of *A. leucotrichus* were submitted to water distillation with a Clevenger-type apparatus (Merck KGaA, Darmstadt, Germany) for 3 h. The final product yielded 2.23% of deep-blue liquid oil. The obtained essential oil was dried over anhydrous sodium sulphate and, after filtration, it was stored at +4 °C.

### 3.3. General Procedure for the Hemi-Synthesis of Compounds ***1**–**4***

Amine derivatives (1.2 mmol, 2,3-diaminomaleonitrile 129.6 mg; *o*-phenylenediamine 129.6 mg; 3-[(2-aminoaryl)amino]dimedone 276 mg; CH_3_-3-[(2-aminoaryl)amino]dimedone 292.8 mg) were added to an ethanolic solution (15 mL) of *A. leucotrichus* essential oil (1 mmol of perillaldehyde, calculated on the basis of 81% of mass, approximately 0.2 mL or 185 mg of the crude oil), under constant agitation at ambient temperature, and the reaction was left for 12 h. Crystals of compounds **1**–**4** were gradually formed in the reaction medium. Precipitates of **1**–**4** were filtered off and washed with ethanol after the required time (as monitored by TLC).

*(S)-2-Amino-3-{[(E)-(4-(prop-1-en-2-yl)cyclohex-1-en-1-yl)methylene]amino}maleonitrile* (**1**). C_14_H_16_N_4_. Brown crystals; yield: 197 mg (82%); m.p. 184–186 °C. ^1^H-NMR (400 MHz, DMSO-d_6_): δ 1.30–1.50 (m, 1H, H-5″), 1.73 (s, 3H, 1‴-CH_3_), 1.77–1.89 (m, 1H, H-5″), 2.07–2.27 (m, 3H, H-4″, H-3″, H-6″), 2.39 (dd, *J* 14.9, 4.0, Hz, 1H, H-3″), 2.56–2.70 (m, 1H, H-6″), 4.66–4.79 (m, 2H, H-2‴), 6.56 (d, *J* 4.7 Hz, 1H, H-2″), 7.48 (s, 2H, 2-NH2), 7.85 (s, 1H, H-1′) ppm. ^13^C-NMR (100 MHz, DMSO-d_6_): δ 21.0 (1‴-CH3), 23.7 (C-6″), 26.8 (C-5″), 31.8 (C-3″), 40.7 (C-4″), 103.8 (C-3), 109.7 (C-2‴), 114.2 (C-4), 114.9 (C-1), 126.2 (C-2), 137.9 (C-1″), 142.6 (C-2″), 149.1 (C-1‴), 158.4 (C-1′) ppm HRMS-ESI^+^: *m*/*z* calcd. for [C_14_H_16_N_4_ + H]^+^: 241.1453; found: 241.1441.

*(S)-2-[4-(Prop-1-en-2-yl)cyclohex-1-en-1-yl]-1H-benzo[d]imidazole* (**2**). C_16_H_18_N_2_. White crystals; yield: 155 mg (65%); m.p. 220–222 °C. ^1^H-NMR (400 MHz, DMSO-d_6_): δ 1.47–1.65 (m, 1H, H-5′), 1.77 (s, 3H, 1″-CH_3_), 1.89–1.99 (m, 1H, H-5′), 2.07–2.49 (m, 4H, H-4′, 2 × H-3′, H-6′), 2.75–2.86 (m, 1H, H-6′), 4.55–5.00 (m, 2H, H-2″), 6.79 (d, *J* 4.0 Hz, 1H, H-2′), 7.07–7.20 (m, 2H, H-7, H-5), 7.42 (d, *J* 7.6 Hz, 1H, H-4), 7.56 (d, *J* 7.6 Hz, 1H, H-6), 12.31 (s, 1H, 1-NH) ppm. ^13^C-NMR (100 MHz, DMSO-d6): δ 21.1 (1″-CH_3_), 25.8 (C-6′), 27.3 (C-5′), 30.9 (C-3′), 40.5 (C-4″), 109.6 (C-2”), 111.2 (C-4), 118.8 (C-6), 121.4 (C-7), 122.3 (C-5), 128.8 (C-1′), 129.2 (C-2′), 135.0 (C-7a), 143.9 (C-3a), 149.2 (C-1”), 158.4 (C-2) ppm. HRMS-ESI^+^: *m*/*z* calcd. for [C_16_H_18_N_2_ + H]^+^: 239.1548; found: 239.1537.

*3,3-Dimethyl-11-[(S)-4-(prop-1-en-2-yl)cyclohex-1-en-1-yl]-2,3,4,5,10,11-hexahydro-1H-dibenzo[b,e][1,4]diazepin-1-one (***3***, mixture of two diastereomers)*. C_24_H_30_N_2_O. Yellowish crystals; yield: 170 mg (47%); m.p. 194–196 °C. ^1^H-NMR (400 MHz, DMSO-d_6_): δ 1.02, 1.03, 1.05 and 1.06 (4s, 6H, 3-CH_3_, 2× diast.), 1.56 and 1.58 (2s, 3H, 1″-CH_3_, 2 × diast.), 1.06–2.25 (m, 7H, H-3′, H-4′, H-5′, H-6′, 2× diast.), 2.02–2.19 (m, 2H, H-2, 2 × diast.), 2.49–2.53 (m, 2H, H-4, 2× diast.), 4.40–4.43 and 4.50–4.53 and 4.54–4.57 and 4.58–4.62 (4m, 2H, H-2″, 2× diast.), 4,81–4.89 (m, 1H, H-11), 5.07 and 5.09 (d, *J* 6.9 Hz 1H, H-2′, 2× diast.), 5.77 and 5.82 (2d, *J* 6.0 Hz, 1H 10-NH, 2× diast.), 6.61–6.77 (m, 3H, H-7, H-8, H-9), 6.90–6.92 and 6.92–6.93 (2m, 1H, H-6, 2× diast.), 8.530 and 8.533 (s, 1H, 5-NH) ppm. ^13^C-NMR (100 MHz, DMSO-d_6_): δ 20.9 and 21.2 (1″-CH_3_, 2× diast.) 27.9 and 28.0 and 29.0 and 29.1 (3-CH_3_, 2× diast.), 26.8 and 27.5 (C-6′, 2× diast.), 27.8 and 28.1 (C-5′,2× diast.), 30.3 and 30.5 (C-3′), 32.2 (C-3), 40.4 and 41.1 (C-4′, 2× diast.), 44.5 (C-4), 50.1 (C-2), 57.0 and 57.8 (C-11, 2× diast.), 109.1 and 109.2 (C-2″, 2× diast.), 110.2 and 110.4 (C-11a, 2× diast.), 119.84 and 119.86 (C-6), 120.17, 120.22, 120.26, 120.39, 120.51 and 120.73 (C-2′, C-7, C-9, 2× diast.), 122.7 and 122.8 (C-8, 2× diast.), 131.9 and 131.8 (C-5a, 2× diast.)), 138.3 and 138.7 (C-9a, 2× diast.)), 139.1 and 139.3 (C-1′, 2× diast.), 149.2 and 149.7 (C-1″, 2× diast.), 154.85 and 154.87 (C-4a, 2× diast.), 192.2 and 192.3 (C-1, C=O, 2× diast.) ppm. HRMS-ESI^+^: *m*/*z* calcd. for [C_24_H_30_N_2_O + H]^+^: 363.2436; found: 363.2419.

*3,3,8-Trimethyl-11-[(S)-4-(prop-1-en-2-yl)cyclohex-1-en-1-yl]-2,3,4,5,10,11-hexahydro-1H-dibenzo[b,e][1,4]diazepin-1-one (***4***, mixture of two diastereomers)*. C_25_H_32_N_2_O. Yellowish crystals; yield: 267 mg (71%); m.p. 201–202 °C. ^1^H-NMR (400 MHz, DMSO-d_6_): δ 1.01, 1.02, 1.04 and 1.06 (4s, 6H, 3-CH_3_, 2× diast.), 1.56 and 1.59 (2s, 3H, 1″-CH_3_, 2× diast.), 1.06–2.16 (m, 7H, H-3′, H-4′, H-5′, H-6′, 2× diast.), 2.11 and 2.13 (2s, 3H, 8-CH_3_, 2× diast.), 2.02–2.19 (m, 2H, H-2, 2× diast.), 2.49–2.51 (m, 2H, H-4, 2× diast.), 4.36–4.40 and 4.48–4.53 and 4.55–4.58 and 4.58–4.62 (4m, 2H, H-2″, 2× diast.), 4.78–4.87 (m, 1H, H-11), 5.06 and 5.09 (d, *J* 6.9 Hz 1H, H-2′, 2× diast.), 5.71 and 5.74 (2d, *J* 6.0 Hz, 1H 10-NH, 2× diast.), 6.41–6.59 (m, 2H, H-6, H-9), 6.81 (dd, *J* 2.8 and 8.4 Hz, 1H, H-7), 8.50 and 8.51 (2s, 1H, 5-NH, 2× diast.) ppm. ^13^C-NMR (100 MHz, DMSO-d_6_): δ 20.77 and 20.79 (8-CH3, 2× diast.), 20.9 and 21.3 (1″-CH_3_, 2× diast.), 27.8 and 28.0 and 29.1 and 29.2 (3-CH_3_, 2× diast.), 26.7 and 27.3 (C-6′, 2× diast.), 27.8 and 28.1 (C-5′,2× diast.), 30.2 and 30.5 (C-3′), 32.1 (C-3), 40.4 and 41.1 (C-4′, 2× diast.), 44.5 (C-4), 50.1 (C-2), 57.0 and 57.7 (C-11, 2× diast.), 109.1 and 109.2 (C-2″, 2× diast.), 109.9 and 110.0 (C-11a, 2× diast.), 120.1 (C-2′), 120.22 and 120.24 (C-7, 2× diast.), 120.50 and 120.52 (C-6, 2× diast.), 120.8 and 120.9 (C-9, 2× diast.), 129.3 and 129.4 (C-8, 2× diast.), 131.5 and 131.6 (C-5a, 2× diast.)), 138.5 and 138.9 (C-9a, 2× diast.)), 139.0 and 139.1 (C-1′, 2× diast.), 149.1 and 149.7 (C-1″, 2× diast.), 154.79 and 154.82 (C-4a, 2× diast.), 191.9 and 192.0 (C-1, C=O, 2× diast.) ppm. HRMS-ESI^+^: *m*/*z* calcd. for [C_25_H_32_N_2_O + H]^+^: 377.2548; found: 377.2577.

## 4. Conclusions

In summary, we used the natural (*S*)-(−)-perillaldehyde of *A. leucotrichus* subsp. *leucotrichus* essential oil as a chiral reagent to stereospecifically prepare imine, imidazole and dibenzo[*b*,*e*][1,4]diazepin-1-ones, which is an efficient, simple and economic hemi-synthetic protocol towards pure chiral compounds. We proved that natural compounds, when present in high amount in essential oil or extracts, can be used as excellent starting materials to prepare new structures in hemi-synthetic processes without their prior isolation and purification. The reported method is advantageously applicable to generate chiral compounds, avoiding the use of expensive chiral catalysts and isolated natural compounds. An arsenal of analytical tools, including 2D NMR, single-crystal X-ray diffraction and chiral HPLC were helpful to identify pure enantiomers and some diastereomeric mixtures. Further hemi-synthetic studies are being conducted on the same and other essential oils’ major compounds to extend the scope and determine the limits of the reported method. Biological screening and comparative studies are also foreseen.

## Supplementary Materials

The Supplementary Materials are available online. Supplementary Information (ESI) available: [NMR spectroscopic data for all the reported compounds **1**–**4**; Chiral-HPLC analysis of compounds **1**–**3**; Specific rotation analysis for pure enantiomers of compounds **1** and **2**; Single-crystal X-ray diffraction data (CIF files and check CIF reports) and additional crystallographic details for compounds **1**–**4**.
