# Supplementary material for: Hemi-Synthesis of Chiral Imine, Benzimidazole and Benzodiazepines from Essential Oil of Ammodaucus leucotrichus subsp. leucotrichus
Source: Molecules. 2019 Mar 10;24(5):975. doi: 10.3390/molecules24050975 (PMC6429316; doi:10.3390/molecules24050975)
Supplement: Supplementary file 1 [file molecules-24-00975-s001.zip › 2_checkCIF.pdf]

checkCIF (full publication check) running

Checking for embedded fcf data in CIF ...
Found embedded fcf data in CIF. Extracting fcf data from uploaded CIF, please wait **. . . .**

checkCIF/PLATON (full publication check)

Structure factors have been supplied for datablock(s) I

THIS REPORT IS FOR GUIDANCE ONLY. IF USED AS PART OF A REVIEW PROCEDURE FOR PUBLICATION, IT SHOULD NOT REPLACE THE EXPERTISE OF AN EXPERIENCED CRYSTALLOGRAPHIC REFEREE.

No syntax errors found. CIF dictionary
Please wait while processing .... Interpreting this report

Structure factor report

Datablock: I

Bond precision: C-C = 0.0050 A Wavelength=0.71073

Cell:	a=8.6559(6)	b=9.8848(7)	c=30.285(2)
	alpha=90	beta=90	gamma=90

Temperature: 150 K

	Calculated	Reported
Volume	2591.2(3)	2591.2(3)
Space group	P 21 21 21	P 21 21 21
Hall group	P 2ac 2ab	P 2ac 2ab
Moiety formula	C16 H18 N2	C16 H18 N2
Sum formula	C16 H18 N2	C16 H18 N2
Mr	238.32	238.32
Dx,g cm-3	1.222	1.222
Z	8	8
Mu (mm-1)	0.072	0.072
F000	1024.0	1024.0
F000'	1024.32	
h,k,lmax	10,11,36	10,11,36
Nref	4740[2723]	4726
Tmin,Tmax	0.987,0.991	0.692,0.745
Tmin'	0.986	

Correction method= # Reported T Limits: Tmin=0.692 Tmax=0.745
AbsCorr = MULTI-SCAN

Data completeness= 1.74/1.00 Theta(max)= 25.342

R(reflections)= 0.0421(3582) wR2(reflections)= 0.0997(4726)

S = 1.050 Npar= 327

The following ALERTS were generated. Each ALERT has the format
test-name_ALERT_alert-type_alert-level.
Click on the hyperlinks for more details of the test.

Alert level B

PLAT111_ALERT_2_B ADDSYM Detects New (Pseudo) Centre of Symmetry . 100 %Fit

Author Response: This is an artifact because the structure needs to be non-centrosymmetric due to the use of a chiral natural product.

PLAT910_ALERT_3_B Missing # of FCF Reflection(s) Below Theta(Min). 12 Note

Alert level C

STRVA01_ALERT_4_C Flack parameter is too small
From the CIF: _refine_ls_abs_structure_Flack -1.700

From the CIF: _refine_ls_abs_structure_Flack_su 1.000
PLAT340_ALERT_3_C Low Bond Precision on C-C Bonds .... 0.005 Ang.
PLAT934_ALERT_3_C Number of (Iobs-Icalc)/SigmaW > 10 Outliers .... 1 Check

Alert level G

PLAT007_ALERT_5_G Number of Unrefined Donor-H Atoms .... 2 Report
PLAT032_ALERT_4_G Std. Uncertainty on Flack Parameter Value High . 1.000 Report
PLAT112_ALERT_2_G ADDSYM Detects New (Pseudo) Symm. Elem b 94 %Fit
And 2 other PLAT112 Alerts
More ...
PLAT113_ALERT_2_G ADDSYM Suggests Possible Pseudo/New Space Group Pbca Check
PLAT720_ALERT_4_G Number of Unusual/Non-Standard Labels .... 29 Note
PLAT791_ALERT_4_G Model has Chirality at C11 (Chiral SPGR) S Verify
PLAT791_ALERT_4_G Model has Chirality at C27 (Chiral SPGR) S Verify
PLAT916_ALERT_2_G Hooft y and Flack x Parameter Values Differ by . 1.77 Check
PLAT978_ALERT_2_G Number C-C Bonds with Positive Residual Density. 3 Info

0 **ALERT level A** = Most likely a serious problem - resolve or explain
2 **ALERT level B** = A potentially serious problem, consider carefully
3 **ALERT level C** = Check. Ensure it is not caused by an omission or oversight
11 **ALERT level G** = General information/check it is not something unexpected

0 ALERT type 1 CIF construction/syntax error, inconsistent or missing data
7 ALERT type 2 Indicator that the structure model may be wrong or deficient
3 ALERT type 3 Indicator that the structure quality may be low
5 ALERT type 4 Improvement, methodology, query or suggestion
1 ALERT type 5 Informative message, check

checkCIF publication errors

Alert level A

PUBL006_ALERT_1_A _publ_requested_journal is missing
e.g. 'Acta Crystallographica Section C'
PUBL008_ALERT_1_A _publ_section_title is missing. Title of paper.
PUBL012_ALERT_1_A _publ_section_abstract is missing.
Abstract of paper in English.
PUBL024_ALERT_1_A The number of authors is greater than 9.
Please specify the role of each of the co-authors
for your paper.

Alert level G

PUBL017_ALERT_1_G The _publ_section_references section is missing or
empty.

4 **ALERT level A** = Data missing that is essential or data in wrong format
1 **ALERT level G** = General alerts. Data that may be required is missing

Publication of your CIF

You should attempt to resolve as many as possible of the alerts in all categories. Often the minor alerts point to easily fixed oversights, errors and omissions in your CIF or refinement strategy, so attention to these fine details can be worthwhile. In order to resolve some of the more serious problems it may be necessary to carry out additional measurements or structure refinements. However, the nature of your study may justify the reported deviations from journal submission requirements and the more serious of these should be commented upon in the discussion or experimental section of a paper or in the "special_details" fields of the CIF. *checkCIF* was carefully designed to identify outliers and unusual parameters, but every test has its limitations and alerts that are not important in a particular case may appear. Conversely, the absence of alerts does not guarantee there are no aspects of the results needing attention. It is up to the individual to critically assess their own results and, if necessary, seek expert advice.

If level A alerts remain, which you believe to be justified deviations, and you intend to submit this CIF for publication in a journal, you should additionally insert an explanation in your CIF using the Validation Reply Form (VRF) below. This will allow your explanation to be considered as part of the review process.

Validation response form

Please find below a validation response form (VRF) that can be filled in and pasted into your CIF.

```
# start Validation Reply Form
_vrf_PUBL006_GLOBAL
;
```

PROBLEM: _publ_requested_journal is missing
RESPONSE: ...
;
_vrf_PUBL008_GLOBAL
;
PROBLEM: _publ_section_title is missing. Title of paper.
RESPONSE: ...
;
_vrf_PUBL012_GLOBAL
;
PROBLEM: _publ_section_abstract is missing.
RESPONSE: ...
;
_vrf_PUBL024_GLOBAL
;
PROBLEM: The number of authors is greater than 9.
RESPONSE: ...
;
end Validation Reply Form

If you wish to submit your CIF for publication in Acta Crystallographica Section C or E, you should upload your CIF via the web. If you wish to submit your CIF for publication in IUCrData you should upload your CIF via the web. If your CIF is to form part of a submission to another IUCr journal, you will be asked, either during electronic submission or by the Co-editor handling your paper, to upload your CIF via our web site.

PLATON version of 19/10/2018; check.def file version of 15/10/2018

Datablock I - ellipsoid plot

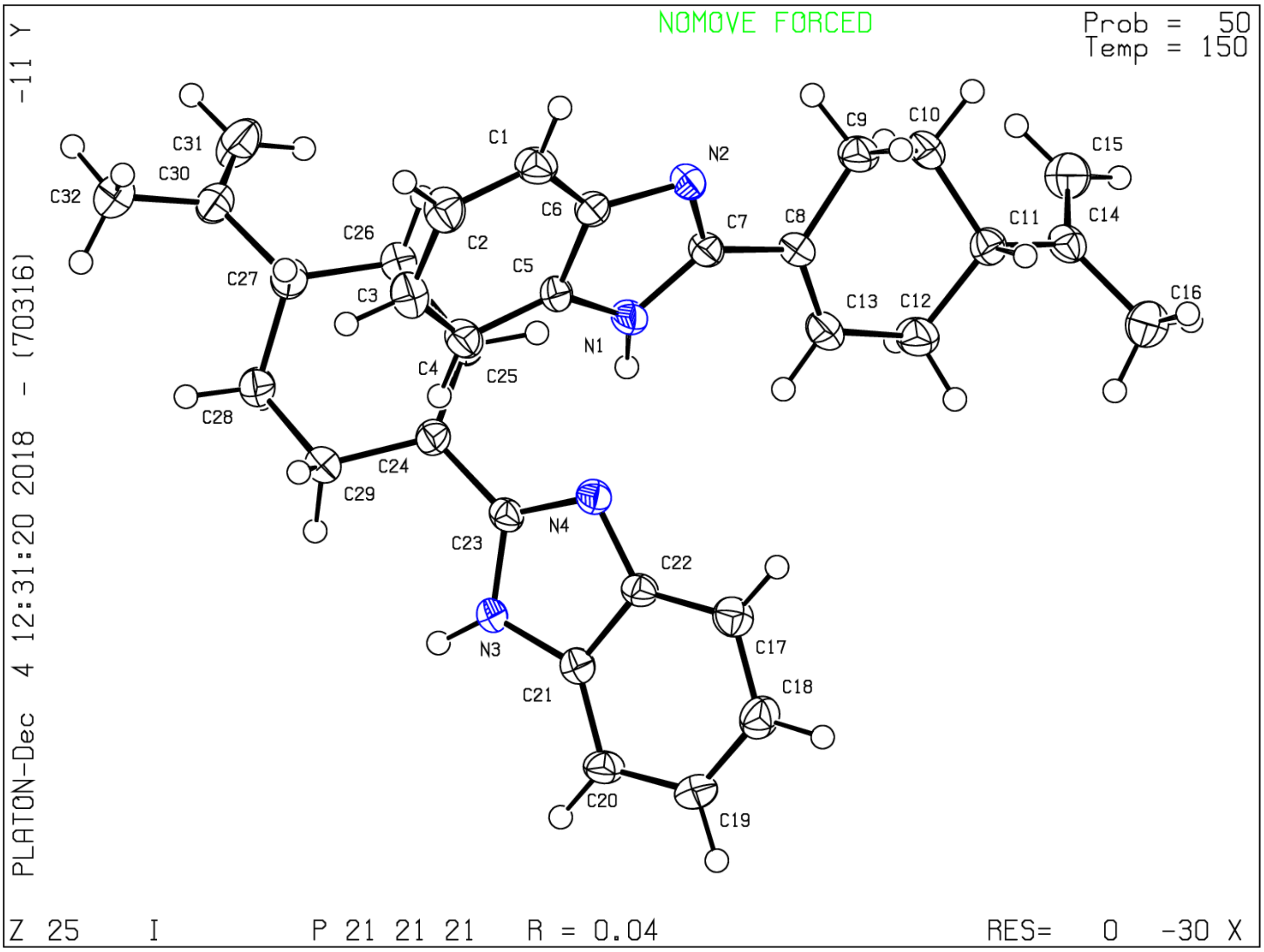

Download CIF editor (publCIF) from the IUCr
Download CIF editor (enCIFer) from the CCDC
Test a new CIF entry
