# Supplementary material for: Hemi-Synthesis of Chiral Imine, Benzimidazole and Benzodiazepines from Essential Oil of Ammodaucus leucotrichus subsp. leucotrichus
Source: Molecules. 2019 Mar 10;24(5):975. doi: 10.3390/molecules24050975 (PMC6429316; doi:10.3390/molecules24050975)
Supplement: Supplementary file 1 [file molecules-24-00975-s001.zip › 3_checkCIF.pdf]

checkCIF (full publication check) running

Checking for embedded fcf data in CIF ...
Found embedded fcf data in CIF. Extracting fcf data from uploaded CIF, please wait . . . . .

checkCIF/PLATON (full publication check)

Structure factors have been supplied for datablock(s) I

THIS REPORT IS FOR GUIDANCE ONLY. IF USED AS PART OF A REVIEW PROCEDURE FOR PUBLICATION, IT SHOULD NOT REPLACE THE EXPERTISE OF AN EXPERIENCED CRYSTALLOGRAPHIC REFEREE.

No syntax errors found.
Please wait while processing ....

CIF dictionary
Interpreting this report

Structure factor report

Datablock: I

```
Bond precision:       C-C = 0.0061 A               Wavelength=0.71073
Cell:        a=11.6743(15)     b=14.0675(18)    c=25.660(4)
             alpha=90          beta=94.342(3)   gamma=90
Temperature: 150 K
                     Calculated                         Reported
Volume               4202.0(10)                         4202.1(10)
Space group          I 2                                I 2
Hall group           I 2y                               I 2y
Moiety formula       C24 H30 N2 O                       C48 H60 N4 O2
Sum formula          C24 H30 N2 O                       C48 H60 N4 O2
Mr                   362.50                             725.00
Dx,g cm-3            1.146                              1.146
Z                    8                                  4
Mu (mm-1)            0.070                              0.070
F000                 1568.0                             1568.0
F000'                1568.56
h,k,lmax             14,16,30                           14,16,30
Nref                 7694[ 4019]                        7592
Tmin,Tmax            0.982,0.994                        0.654,0.745
Tmin'                0.982
Correction method= # Reported T Limits: Tmin=0.654 Tmax=0.745
AbsCorr = MULTI-SCAN
Data completeness= 1.89/0.99      Theta(max)= 25.343
R(reflections)= 0.0525( 6365)        wR2(reflections)= 0.1208( 7592)
S = 1.124              Npar= 504
```

The following ALERTS were generated. Each ALERT has the format
test-name_ALERT_alert-type_alert-level.
Click on the hyperlinks for more details of the test.

Alert level B

PLAT201_ALERT_2_B Isotropic non-H Atoms in Main Residue(s) .... 2 Report

Author Response: This alert arises due to the isotropic treatment used to model the disordered terminal methyl groups belonging to both individual molecules composing the asymmetric unit of the compound.

PLAT910_ALERT_3_B Missing # of FCF Reflection(s) Below Theta(Min). 16 Note

Alert level C

STRVA01_ALERT_2_C Chirality of atom sites is inverted?

From the CIF: _refine_ls_abs_structure_Flack 1.700
From the CIF: _refine_ls_abs_structure_Flack_su 0.900
PLAT220_ALERT_2_C Non-Solvent Resd 1 C Ueq(max)/Ueq(min) Range 3.7 Ratio
PLAT220_ALERT_2_C Non-Solvent Resd 2 C Ueq(max)/Ueq(min) Range 3.9 Ratio
PLAT222_ALERT_3_C Non-Solv. Resd 1 H Uiso(max)/Uiso(min) Range 4.2 Ratio
PLAT340_ALERT_3_C Low Bond Precision on C-C Bonds .... 0.00613 Ang.
PLAT907_ALERT_2_C Flack x > 0.5, Structure Needs to be Inverted? . 1.70 Check
PLAT911_ALERT_3_C Missing FCF Refl Between Thmin & STh/L= 0.600 4 Report

---

Alert level G
PLAT002_ALERT_2_G Number of Distance or Angle Restraints on AtSite 8 Note
PLAT032_ALERT_4_G Std. Uncertainty on Flack Parameter Value High . 0.900 Report
PLAT042_ALERT_1_G Calc. and Reported MoietyFormula Strings Differ Please Check
PLAT045_ALERT_1_G Calculated and Reported Z Differ by a Factor ... 2.00 Check
PLAT111_ALERT_2_G ADDSYM Detects New (Pseudo) Centre of Symmetry . 96 %Fit
PLAT112_ALERT_2_G ADDSYM Detects New (Pseudo) Symm. Elem c 88 %Fit
PLAT113_ALERT_2_G ADDSYM Suggests Possible Pseudo/New Space Group I2/c Check
PLAT172_ALERT_4_G The CIF-Embedded .res File Contains DFIX Records 1 Report
PLAT300_ALERT_4_G Atom Site Occupancy of C48 Constrained at 0.6 Check
And 15 other PLAT300 Alerts
More ...
PLAT301_ALERT_3_G Main Residue Disorder ....(Resd 1) 4% Note
PLAT301_ALERT_3_G Main Residue Disorder ....(Resd 2) 4% Note
PLAT791_ALERT_4_G Model has Chirality at C15 (Chiral SPGR) S Verify
And 3 other PLAT791 Alerts
More ...
PLAT860_ALERT_3_G Number of Least-Squares Restraints .... 5 Note
PLAT916_ALERT_2_G Hooft y and Flack x Parameter Values Differ by . 0.11 Check
PLAT978_ALERT_2_G Number C-C Bonds with Positive Residual Density. 12 Info

---

0 **ALERT level A** = Most likely a serious problem - resolve or explain
2 **ALERT level B** = A potentially serious problem, consider carefully
7 **ALERT level C** = Check. Ensure it is not caused by an omission or oversight
33 **ALERT level G** = General information/check it is not something unexpected

2 ALERT type 1 CIF construction/syntax error, inconsistent or missing data
11 ALERT type 2 Indicator that the structure model may be wrong or deficient
7 ALERT type 3 Indicator that the structure quality may be low
22 ALERT type 4 Improvement, methodology, query or suggestion
0 ALERT type 5 Informative message, check

---

checkCIF publication errors

Alert level A
PUBL006_ALERT_1_A _publ_requested_journal is missing
e.g. 'Acta Crystallographica Section C'
PUBL008_ALERT_1_A _publ_section_title is missing. Title of paper.
PUBL012_ALERT_1_A _publ_section_abstract is missing.
Abstract of paper in English.
PUBL024_ALERT_1_A The number of authors is greater than 9.
Please specify the role of each of the co-authors
for your paper.

Alert level G
PUBL017_ALERT_1_G The _publ_section_references section is missing or
empty.

---

4 **ALERT level A** = Data missing that is essential or data in wrong format
1 **ALERT level G** = General alerts. Data that may be required is missing

---

Publication of your CIF

You should attempt to resolve as many as possible of the alerts in all categories. Often the minor alerts point to easily fixed oversights, errors and omissions in your CIF or refinement strategy, so attention to these fine details can be worthwhile. In order to resolve some of the more serious problems it may be necessary to carry out additional measurements or structure refinements. However, the nature of your study may justify the reported deviations from journal submission requirements and the more serious of these should be commented upon in the discussion or experimental section of a paper or in the "special_details" fields of the CIF. *checkCIF* was carefully designed to identify outliers and unusual parameters, but every test has its limitations and alerts that are not important in a particular case may appear. Conversely, the absence of

alerts does not guarantee there are no aspects of the results needing attention. It is up to the individual to critically assess their own results and, if necessary, seek expert advice.

If level A alerts remain, which you believe to be justified deviations, and you intend to submit this CIF for publication in a journal, you should additionally insert an explanation in your CIF using the Validation Reply Form (VRF) below. This will allow your explanation to be considered as part of the review process.

Validation response form

Please find below a validation response form (VRF) that can be filled in and pasted into your CIF.

```
# start Validation Reply Form
_vrf_PUBL006_GLOBAL
;
PROBLEM: _publ_requested_journal is missing
RESPONSE: ...
;
_vrf_PUBL008_GLOBAL
;
PROBLEM: _publ_section_title is missing. Title of paper.
RESPONSE: ...
;
_vrf_PUBL012_GLOBAL
;
PROBLEM: _publ_section_abstract is missing.
RESPONSE: ...
;
_vrf_PUBL024_GLOBAL
;
PROBLEM: The number of authors is greater than 9.
RESPONSE: ...
;
# end Validation Reply Form
```

If you wish to submit your CIF for publication in Acta Crystallographica Section C or E, you should upload your CIF via the web. If you wish to submit your CIF for publication in IUCrData you should upload your CIF via the web. If your CIF is to form part of a submission to another IUCr journal, you will be asked, either during electronic submission or by the Co-editor handling your paper, to upload your CIF via our web site.

PLATON version of 19/10/2018; check.def file version of 15/10/2018

Datablock I - ellipsoid plot

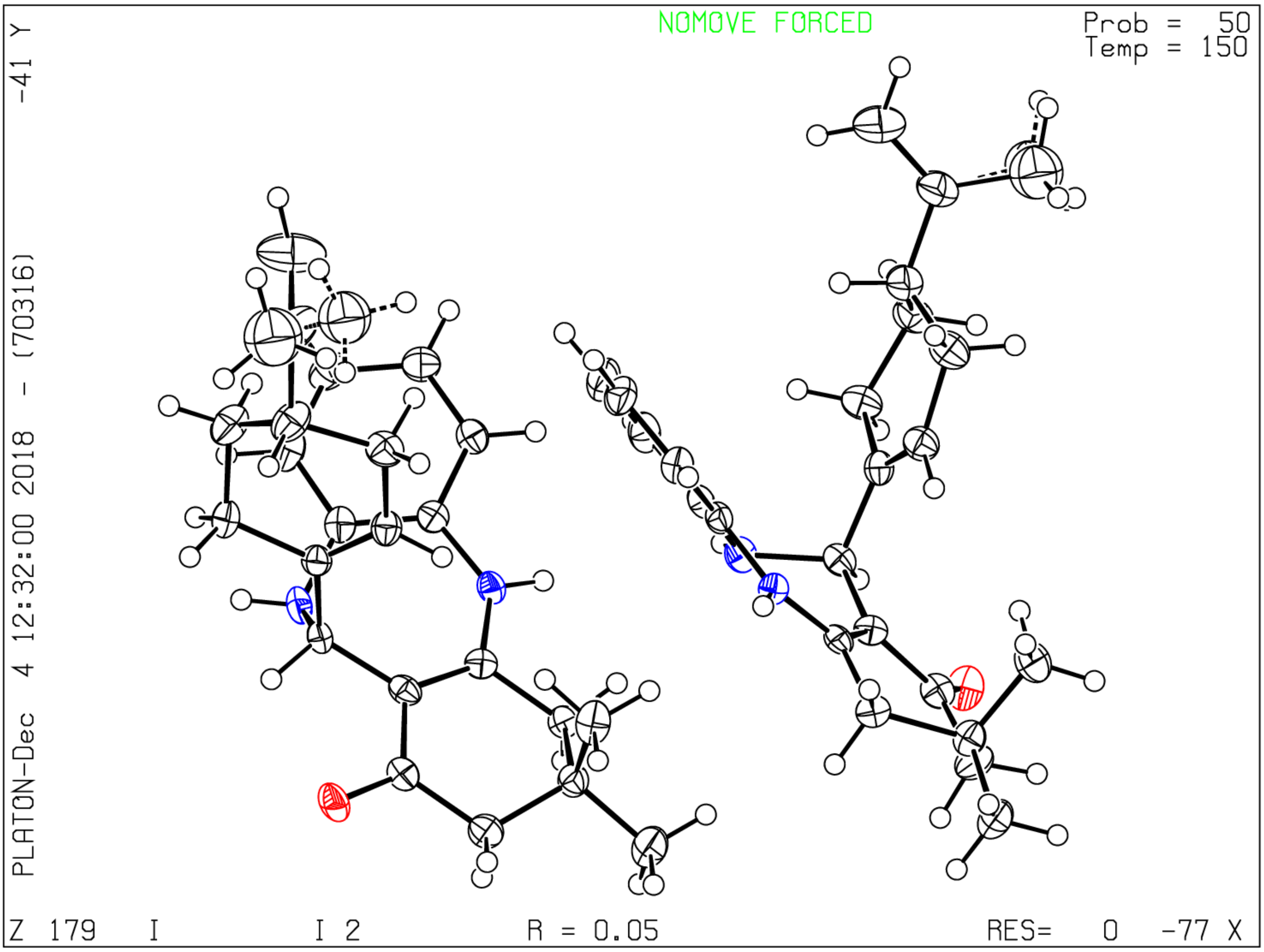

Download CIF editor (publCIF) from the IUCr
Download CIF editor (enCIFer) from the CCDC
Test a new CIF entry
