# Supplementary material for: Hemi-Synthesis of Chiral Imine, Benzimidazole and Benzodiazepines from Essential Oil of Ammodaucus leucotrichus subsp. leucotrichus
Source: Molecules. 2019 Mar 10;24(5):975. doi: 10.3390/molecules24050975 (PMC6429316; doi:10.3390/molecules24050975)
Supplement: Supplementary file 1 [file molecules-24-00975-s001.zip › CCDC Depository Request.pdf]

From: **CCDC Deposit** deposit_reply@ccdc.cam.ac.uk
Subject: CCDC Depository Request
Date: 4 December 2018 at 12:50
To: Filipe Almeida Paz filipe.paz@ua.pt

CD

Dear Depositor,

Thank you for depositing your crystal structure(s) via the joint CCDC/FIZ Karlsruhe deposition service.

The data have been assigned the following deposition numbers which can either be quoted as CCDC Numbers or CSD Numbers. A CCDC Number is usually quoted for an organic or metal-organic structure, whereas a CSD Number is usually quoted for an inorganic structure.

CCDC XXXXXXX-YYYYYYY (generally used for organic and metal-organic structures)

CSD XXXXXXX-YYYYYYY (generally used for inorganic structures)

CCDC 1883076-1883079

Summary of Data CCDC 1883076

Compound Name:
Formula: C14 H16 N4
Unit Cell Parameters: a 6.1702(13) b 7.1689(15) c 15.438(3) P21

Summary of Data CCDC 1883077

Compound Name:
Formula: C16 H18 N2
Unit Cell Parameters: a 8.6559(6) b 9.8848(7) c 30.285(2) P212121

Summary of Data CCDC 1883078

Compound Name:
Formula: C24 H30 N2 O1
Unit Cell Parameters: a 11.6743(15) b 14.0675(18) c 25.660(4) I2

Summary of Data CCDC 1883079

Compound Name:
Formula: C25 H32 N2 O1
Unit Cell Parameters: a 11.6925(14) b 14.2324(16) c 26.184(4) I2

After publication your data will be made available through our joint Access Structures service. In addition, organic and metal-organic experimental structures will be curated into the Cambridge Structural Database and inorganic experimental structures will be curated into the Inorganic Crystal Structure Database.

If you selected “Publish in a Database” your data will be immediately published through our joint Access Structures service.

Please note, if any of these structures are not published within one year from today and we cannot contact you to discuss the matter, then we may publish the data directly through the CSD as a *CSD Communication* or the ICSD as an *ICSD Communication*.

If we have any queries relating to the data then we may contact you later.

Kind Regards,

The CCDC and FIZ Karlsruhe Deposition Teams

Email: deposit@ccdc.cam.ac.uk
The Cambridge Crystallographic Data Centre
https://www.ccdc.cam.ac.uk
For more information about CSD Communications see:
https://www.ccdc.cam.ac.uk/Community/Depositastructure/CSDCommunications/
FIZ Karlsruhe
www.fiz-karlsruhe.de

The CCDC and FIZ Karlsruhe are delighted to be working together on shared deposition and access services for crystallographic data across all domains of chemistry

More details can be found in our press release: https://www.ccdc.cam.ac.uk/News/List/2018-07-new-joint-services/
