# Supplementary material for: Hemi-Synthesis of Chiral Imine, Benzimidazole and Benzodiazepines from Essential Oil of Ammodaucus leucotrichus subsp. leucotrichus
Source: Molecules. 2019 Mar 10;24(5):975. doi: 10.3390/molecules24050975 (PMC6429316; doi:10.3390/molecules24050975)
Supplement: Supplementary file 1 [file molecules-24-00975-s001.zip › molecules-444652-supplementary.pdf]

Supplementary Material

Table of Contents:

Gas chromatography–mass spectrometry (GC/MS) and identification of the essential oil (*Ammodaucus leucotrichus* subsp. *leucotrichus*) constituents 2

Figure S1. GC-MS profile of *Ammodaucus leucotrichus* subsp. *leucotrichus* essential oil 2

Nuclear Magnetic Resonance spectra and data of compound 1-4 3

Figure S2. ^{1}H NMR spectrum of compound **1** (400 MHz) 5

Figure S3. ^{13}C NMR spectrum of compound **1** (100 MHz) 5

Figure S4. HSQC NMR spectrum of compound **1** 6

Figure S5. HMBC NMR spectrum of compound **1** 6

Figure S6. ^{1}H NMR spectrum of compound **2** (400 MHz) 7

Figure S7. ^{13}C NMR spectrum of compound **2** (100 MHz) 7

Figure S8. HSQC NMR spectrum of compound **2** 8

Figure S9. HMBC NMR spectrum of compound **2** 8

Figure S10. ^{1}H NMR spectrum of compound **3** (400 MHz) 9

Figure S11. ^{13}C NMR spectrum of compound **3** (100 MHz) 9

Figure S12. HSQC NMR spectrum of compound **3** 10

Figure S13. HMBC NMR spectrum of compound **3** 10

Figure S14. ^{1}H NMR spectrum of compound **4** (400 MHz) 11

Figure S15. ^{13}C NMR spectrum of compound **4** (100 MHz) 11

Chiral-HPLC analysis of compounds **1-4** 12

Figure S16. Chiral-HPLC analysis of compound **1** 12

Figure S17. Chiral-HPLC analysis of compound **2** 12

Figure S18. Chiral-HPLC analysis of compound **3** 13

Specific rotation analysis for pure enantiomers of compound 1 and 2 13

Single-Crystal X-Ray Diffraction Studies for compounds **1-4** 15

References 16

Gas chromatography–mass spectrometry (GC/MS) and identification of the essential oil (*Ammodaucus leucotrichus* subsp. *leucotrichus*) constituents:

GC/MS analysis were performed in a Hewlett-Packard computerized system comprising a 6890 gas chromatograph coupled to a 5973A mass spectrometer, using a fused-silica capillary column HP5-MS (30 m × 0.25 mm,i.d, 0.25 µm film thickness). GC/MS spectra were obtained using the following conditions: carrier gas He, with a flow rate of 0.7 mL/min in the splitless mode; injection volume: 0.1 µL; injection temperature 250°C; the oven temperature was held at 45°C for 6 min and increased from 45°C to 220°C at a rate of 2°C/min and held at 220°C for 14 min. An electron ionization system with ionization energy of 70 eV was used over a scan range of 29-550 m/z. The identification of the oil constituents was based on a comparison of their GC/MS Kovats retention indices (RI), determined with reference to a homologous series of C6–C28 n-alkanes with those of literature. Identification was confirmed by comparison of their mass spectral fragmentation patterns with those stored in the data bank mass spectra (Wiley 7N and NIST 2007 libraries) and with mass spectra literature data. The percentages of the constituents were calculated by integration peak areas using the Chemstation software.

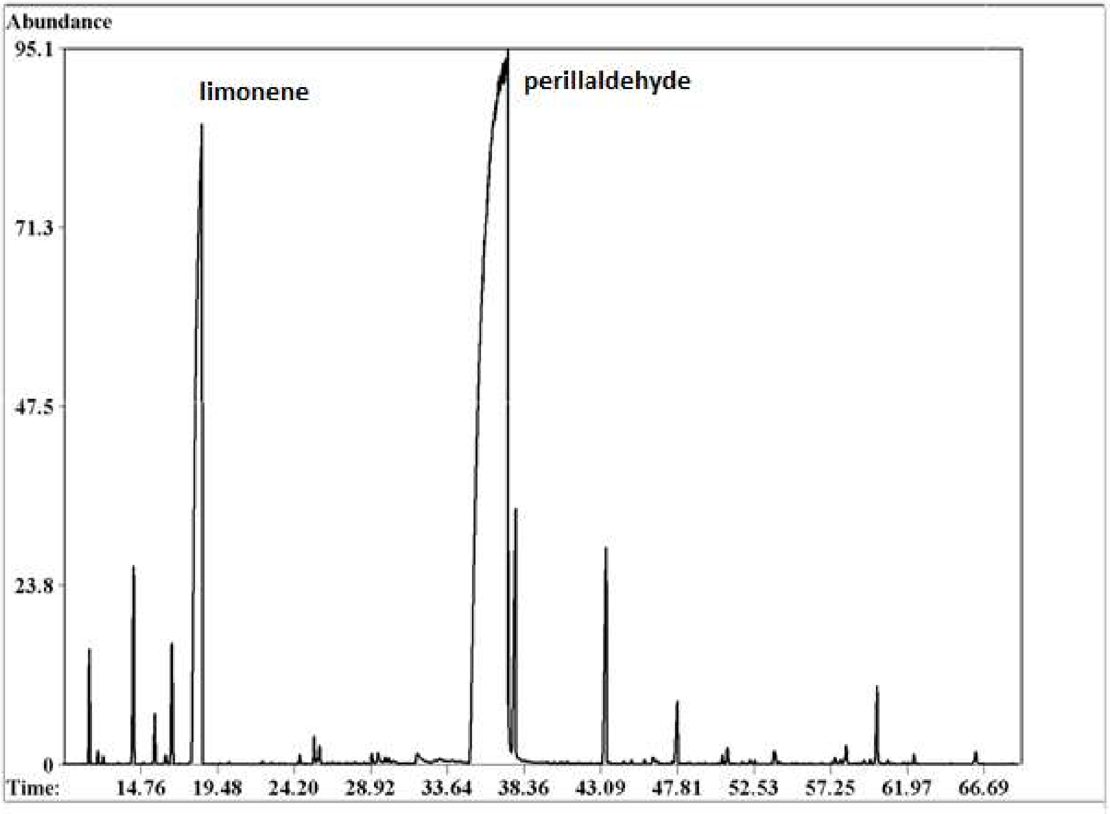

Figure S1. GC-MS profile of *Ammodaucus leucotrichus* subsp. *leucotrichus* essential oil.

Nuclear Magnetic Resonance spectra and data of compound 1-4:

(*S*)-2-Amino-3-{[(*E*)-(4-(prop-1-en-2-yl)cyclohex-1-en-1-yl)methylene]amino}maleonitrile (1):

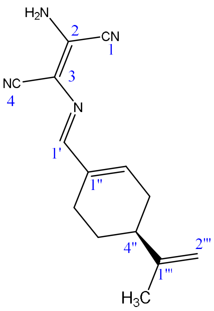

$C_{14}H_{16}N_4$. Brown crystals; yield: 197 mg (82%); mp 184–186 °C. ^{1}H NMR (400 MHz, DMSO-d_6): δ 1.30-1.50 (m, 1H, H-5''), 1.73 (s, 3H, 1'''-CH_3), 1.77-1.89 (m, 1H, H-5''), 2.07-2.27 (m, 3H, H-4'', H-3'', H-6''), 2.39 (dd, *J* 14.9, 4.0, Hz, 1H, H-3''), 2.56-2.70 (m, 1H, H-6''), 4.66-4.79 (m, 2H, H-2'''), 6.56 (d, *J* 4.7 Hz, 1H, H-2''), 7.48 (s, 2H, 2-NH_2), 7.85 (s, 1H, H-1') ppm. ^{13}C NMR (100 MHz, DMSO-d_6): δ 21.0 (1'''-CH_3), 23.7 (C-6''), 26.8 (C-5''), 31.8 (C-3''), 40.7 (C-4''), 103.8 (C-3), 109.7 (C-2'''), 114.2 (C-4), 114.9 (C-1), 126.2 (C-2), 137.9 (C-1''), 142.6 (C-2''), 149.1 (C-1'''), 158.4 (C-1') ppm HRMS-ESI$^+$: m/z calcd for $[C_{14}H_{16}N_4+H]^+$: 241.1453; found: 241.1441.

(*S*)-2-(4-(Prop-1-en-2-yl)cyclohex-1-en-1-yl)-1*H*-benzo[*d*]imidazole (2):

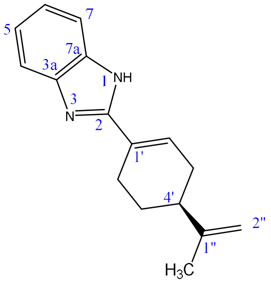

$C_{16}H_{18}N_2$. White crystals; yield: 155 mg (65%); mp 220–222°C. ^{1}H NMR (400 MHz, DMSO-d_6): δ 1.47-1.65 (m, 1H, H-5'), 1.77 (s, 3H, 1''-CH_3), 1.89-1.99 (m, 1H, H-5'), 2.07-2.49 (m, 4H, H-4', 2 x H-3', H-6'), 2.75-2.86 (m, 1H, H-6'), 4.55-5.00 (m, 2H, H-2''), 6.79 (d, *J* 4.0 Hz, 1H, H-2'), 7.07-7.20 (m, 2H, H-7, H-5), 7.42 (d, *J* 7.6 Hz, 1H, H-4), 7.56 (d, *J* 7.6 Hz, 1H, H-6), 12.31 (s, 1H, 1-N*H*) ppm. ^{13}C NMR (100 MHz, DMSO-d_6): δ 21.1 (1''-CH_3), 25.8 (C-6'), 27.3 (C-5'), 30.9 (C-3'), 40.5 (C-4''), 109.6 (C-2''), 111.2 (C-4), 118.8 (C-6), 121.4 (C-7), 122.3 (C-5), 128.8 (C-1'), 129.2 (C-2'), 135.0 (C-7a), 143.9 (C-3a), 149.2 (C-1''), 158.4 (C-2) ppm. HRMS-ESI$^+$: m/z calcd for $[C_{16}H_{18}N_2+H]^+$: 239.1548; found: 239.1537.

3,3-Dimethyl-11-[(*S*)-4-(prop-1-en-2-yl)cyclohex-1-en-1-yl]-2,3,4,5,10,11-hexahydro-1*H*-dibenzo[*b,e*][1,4]diazepin-1-one (3):

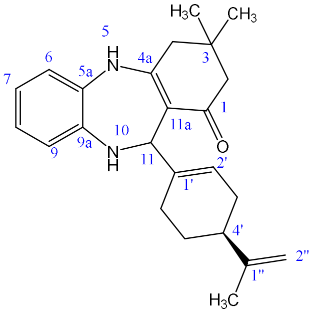

$C_{24}H_{30}N_2O$. Yellowish crystals; yield: 170 mg (47%); mp 194–196 °C. ^{1}H NMR (400 MHz, DMSO-d_6): δ 1.02, 1.03, 1.05 and 1.06 (4s, 6H, 3-CH_3, 2 x diast.), 1.56 and 1.58 (2s, 3H, 1''-CH_3, 2 x diast.), 1.06-2.25 (m, 7H, H-3', H-4', H-5', H-6', 2 x diast.), 2.02-2.19 (m, 2H, H-2, 2 x diast.), 2.49-2.53 (m, 2H, H-4, 2 x diast.), 4.40-4.43 and 4.50-4.53 and 4.54-4.57 and 4.58-4.62 (4m, 2H, H-2'', 2x diast.), 4,81-4.89 (m, 1H, H-11), 5.07 and 5.09 (d, *J* 6.9 Hz 1H, H-2', 2 x diast.), 5.77 and 5.82 (2d, *J* 6.0 Hz, 1H 10-N*H*, 2 x diast.), 6.61-6.77 (m, 3H, H-7, H-8, H-9), 6.90-6.92 and 6.92-6.92 (2m, 1H, H-6, 2 x diast.), 8.530 and 8.533 (s, 1H, 5-N*H*) ppm. ^{13}C NMR (100 MHz, DMSO-d_6): δ 20.9 and 21.2 (1''-CH_3, 2 x diast.) 27.9 and 28.0 and 29.0 and 29.1 (3-CH_3, 2 x diast.), 26.8 and 27.5 (C-6', 2 x diast.), 27.8 and 28.1 (C-5',2 x diast.), 30.3 and 30.5 (C-3'), 32.2 (C-3), 40.4 and 41.1 (C-4', 2 x diast.), 44.5 (C-4), 50.1 (C-2), 57.0 and 57.8 (C-11, 2 x diast.), 109.1 and 109.2 (C-2'', 2 x diast.), 110.2 and 110.4 (C-11a, 2 x diast.), 119.84 and 119.86 (C-6), 120.17, 120.22, 120.26, 120.39, 120.51 and 120.73 (C-2', C-7, C-9, 2 x diast.), 122.7 and 122.8 (C-8, 2 x diast.), 131.9 and 131.8 (C-5a, 2 x diast.), 138.3 and 138.7 (C-9a, 2 x diast.), 139.1 and

139.3 (C-1′, 2 x diast.), 149.2 and 149.7 (C-1′′, 2 x diast.), 154.85 and 154.87 (C-4a, 2 x diast.), 192.2 and 192.3 (C-1, C=O, 2 x diast.) ppm. HRMS-ESI$^+$: m/z calcd for $[C_{24}H_{30}N_2O+H]^+$: 363.2436; found: 363.2419.

3,3,8-Trimethyl-11-[(*S*)-4-(prop-1-en-2-yl)cyclohex-1-en-1-yl]-2,3,4,5,10,11-hexahydro-1*H*-dibenzo[*b,e*][1,4]diazepin-1-one (4):

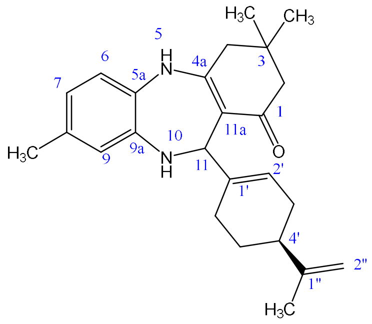

$C_{25}H_{32}N_2O$. Yellowish crystals; yield: 267 mg (71%); mp 201–202 °C. ^{1}HNMR (400 MHz, DMSO-d_6): δ 1.01, 1.02, 1.04 and 1.06 (4s, 6H, 3-CH_3, 2 x diast.), 1.56 and 1.59 (2s, 3H, 1′′-CH_3, 2 x diast.), 1.06-2.16 (m, 7H, H-3′, H-4′, H-5′, H-6′, 2 x diast.), 2.11 and 2.13 (2s, 3H, 8-CH_3, 2 x diast.), 2.02-2.19 (m, 2H, H-2, 2 x diast.), 2.49-2.51 (m, 2H, H-4, 2 x diast.), 4.36-4.40 and 4.48-4.53 and 4.55-4.58 and 4.58-4.62 (4m, 2H, H-2′′, 2x diast.), 4.78-4.87 (m, 1H, H-11), 5.06 and 5.09 (d, *J* 6.9 Hz, 1H, H-2′, 2 x diast.), 5.71 and 5.74 (2d, *J* 6.0 Hz, 1H 10-N*H*, 2 x diast.), 6.41-6.59 (m, 2H, H-6, H-9), 6.81 (dd, *J* 2.8 and 8.4 Hz, 1H, H-7), 8.50 and 8.51 (2s, 1H, 5-N*H,* 2 x diast.) ppm. ^{13}C NMR (100 MHz, DMSO-d_6): δ 20.77 and 20.79 (8-*C*H$_3$, 2 x diast.), 20.9 and 21.3 (1′′-*C*H$_3$, 2 x diast.), 27.8 and 28.0 and 29.1 and 29.2 (3-*C*H$_3$, 2 x diast.), 26.7 and 27.3 (C-6′, 2 x diast.), 27.8 and 28.1 (C-5′,2 x diast.), 30.2 and 30.5 (C-3′), 32.1 (C-3), 40.4 and 41.1 (C-4′, 2 x diast.), 44.5 (C-4), 50.1 (C-2), 57.0 and 57.7 (C-11, 2 x diast.), 109.1 and 109.2 (C-2′′, 2 x diast.), 109.9 and 110.0 (C-11a, 2 x diast.), 120.1 (C-2′), 120.22 and 120.24 (C-7, 2 x diast.), 120.50 and 120.52 (C-6, 2 x diast.), 120.8 and 120.9 (C-9, 2 x diast.), 129.3 and 129.4 (C-8, 2 x diast.), 131.5 and 131.6 (C-5a, 2 x diast.), 138.5 and 138.9 (C-9a, 2 x diast.), 139.0 and 139.1 (C-1′, 2 x diast.), 149.1 and 149.7 (C-1′′, 2 x diast.), 154.79 and 154.82 (C-4a, 2 x diast.), 191.9 and 192.0 (C-1, C=O, 2 x diast.) ppm. HRMS-ESI$^+$: m/z calcd for $[C_{25}H_{32}N_2O+H]^+$: 377.2548; found: 377.2577.

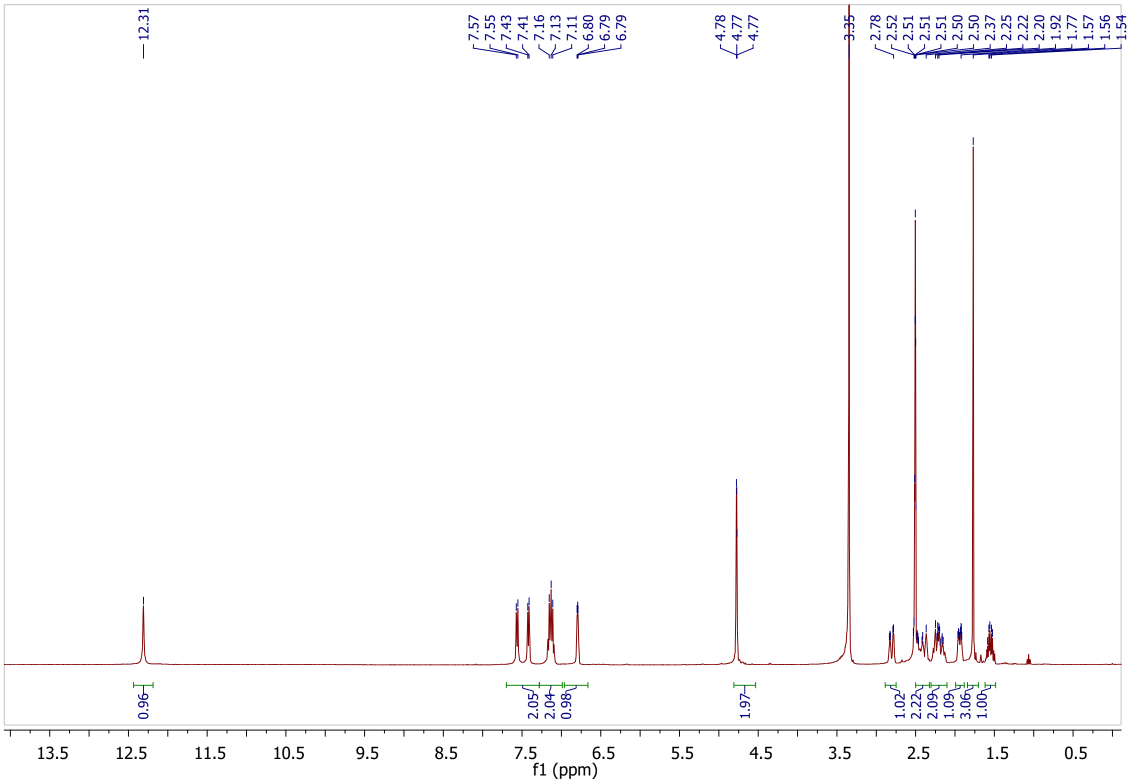

Figure S2. ^{1}H NMR spectrum of compound **1** (400 MHz).

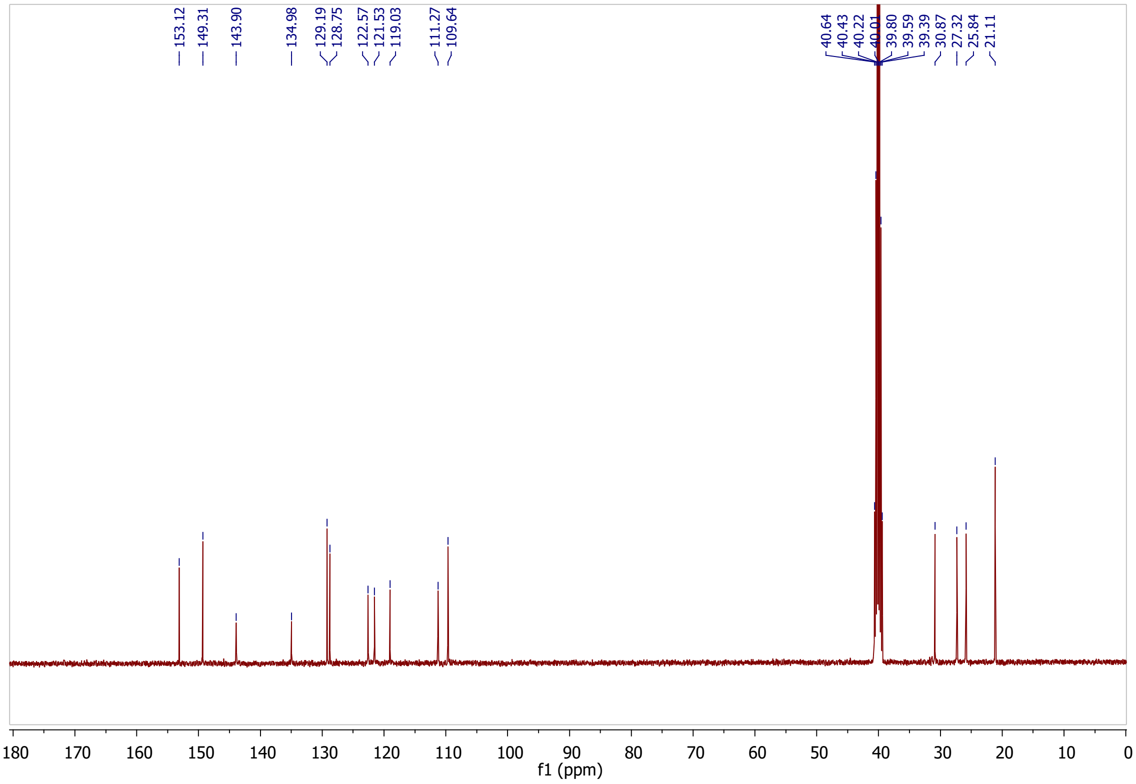

Figure S3. ^{13}C NMR spectrum of compound **1** (100 MHz).

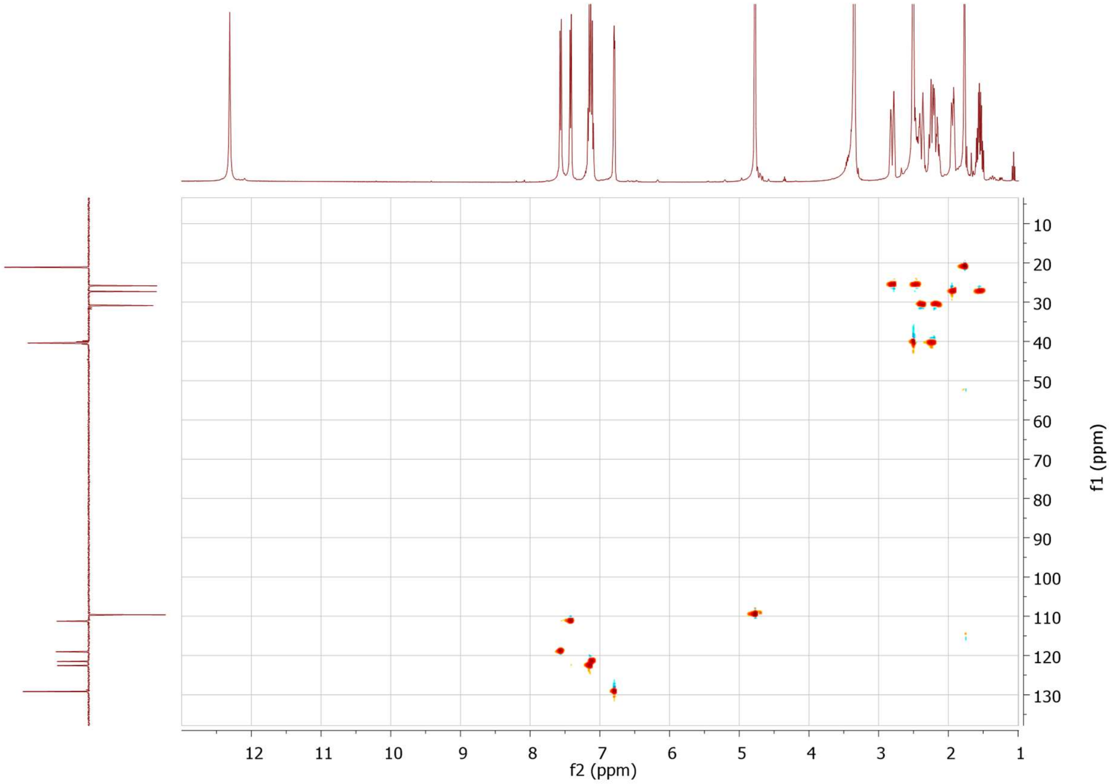

Figure S4. HSQC NMR spectrum of compound **1**.

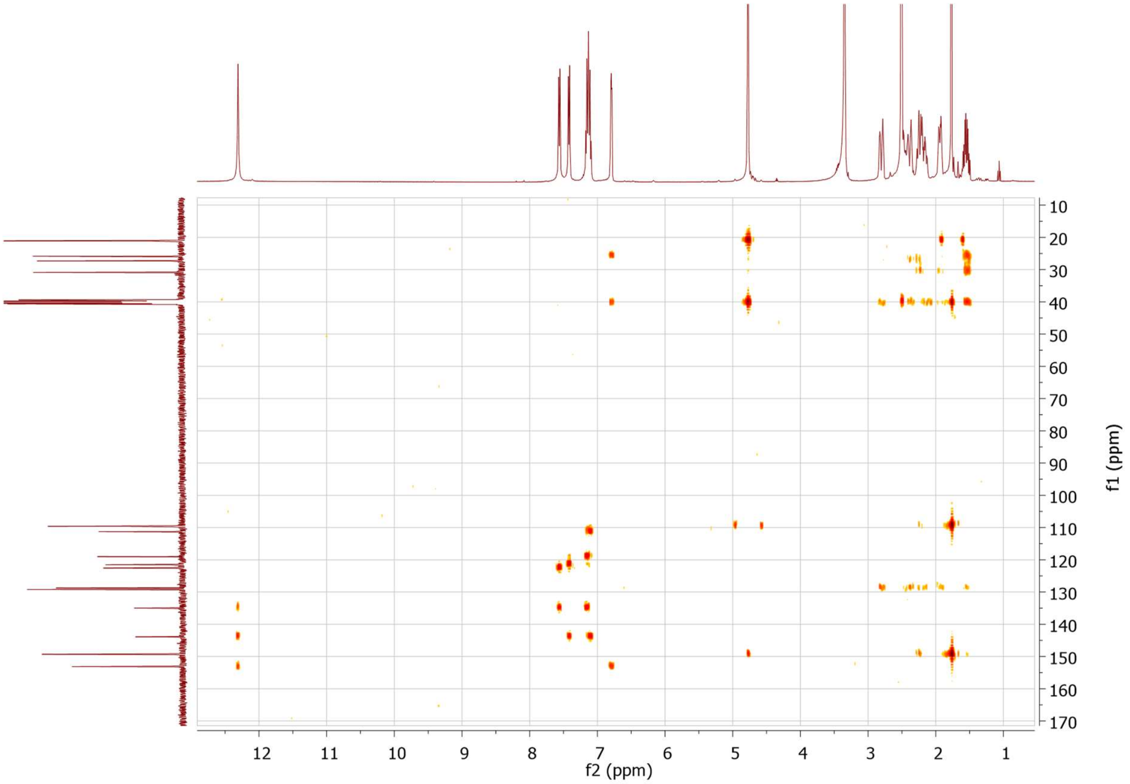

Figure S5. HMBC NMR spectrum of compound **1**.

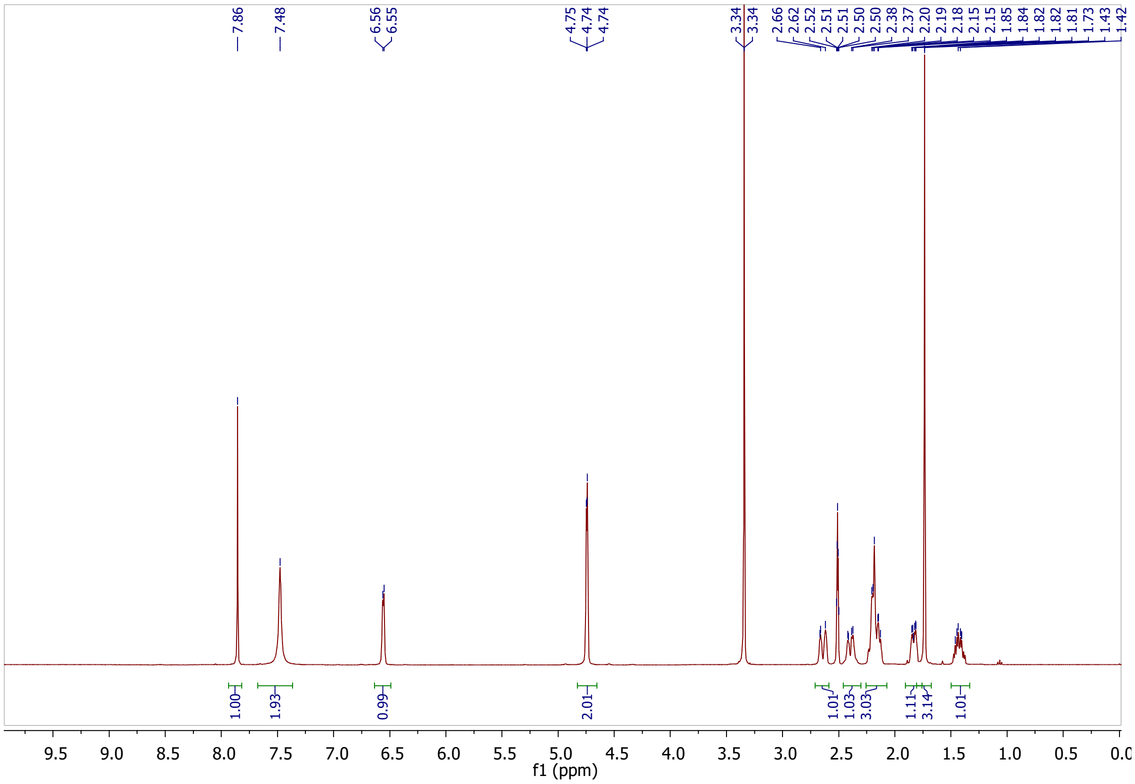

Figure S6. ^{1}H NMR spectrum of compound **2** (400 MHz).

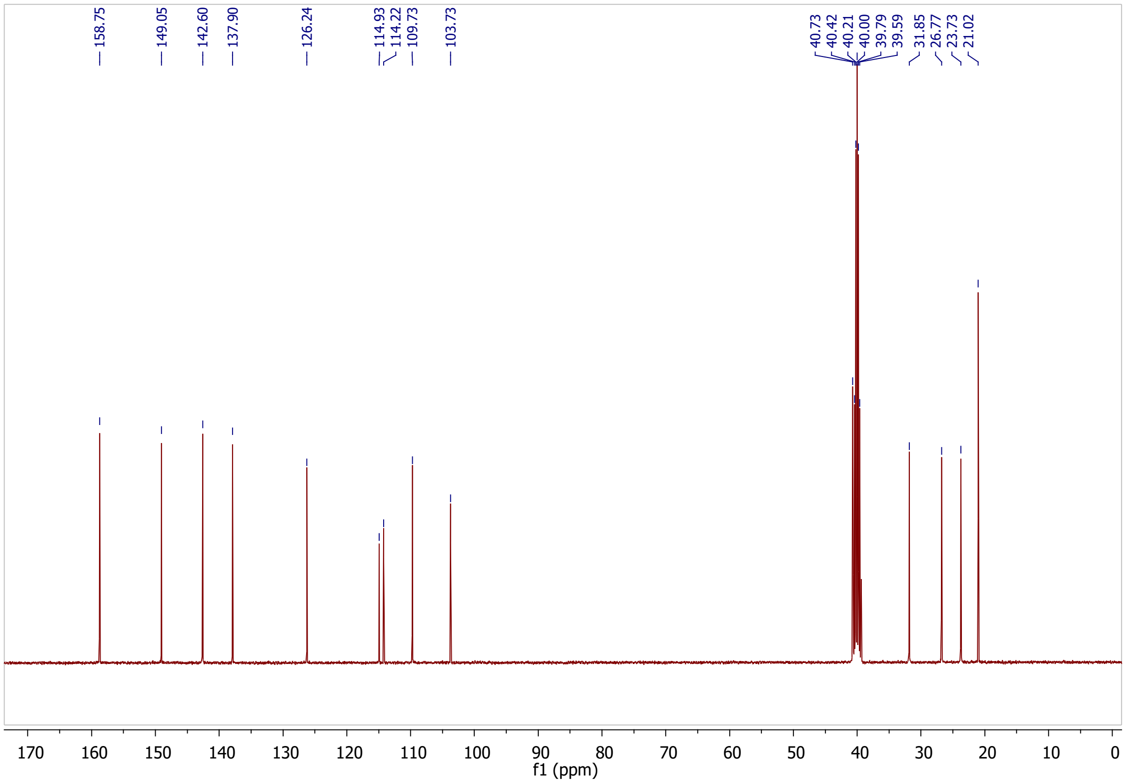

Figure S7. ^{13}C NMR spectrum of compound **2** (100 MHz).

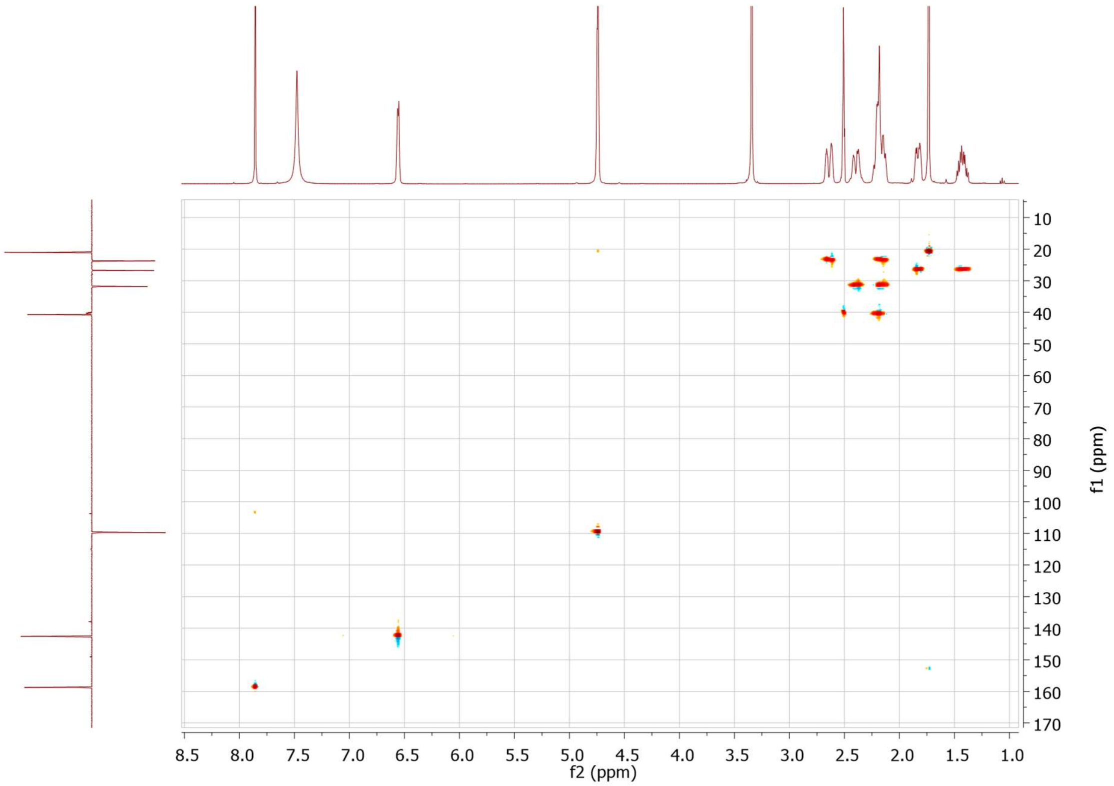

Figure S8. HSQC NMR spectrum of compound **2**.

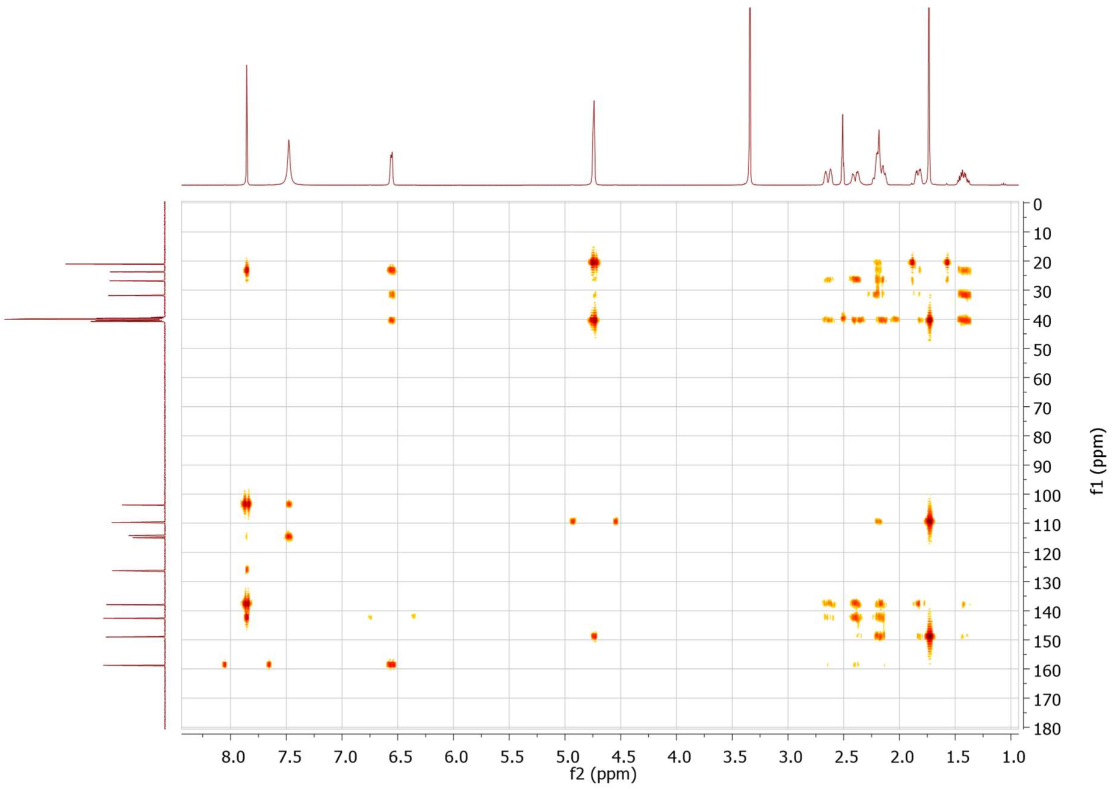

Figure S9. HMBC NMR spectrum of compound **2**.

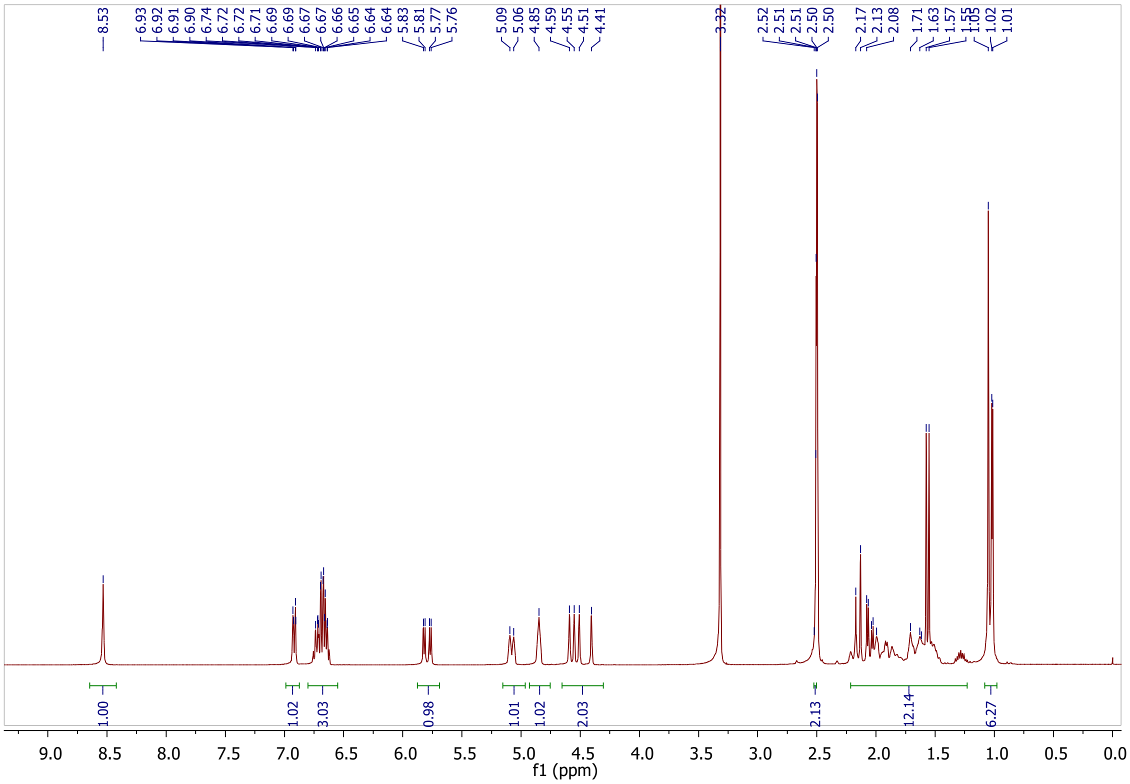

Figure S10. ^{1}H NMR spectrum of compound **3** (400 MHz).

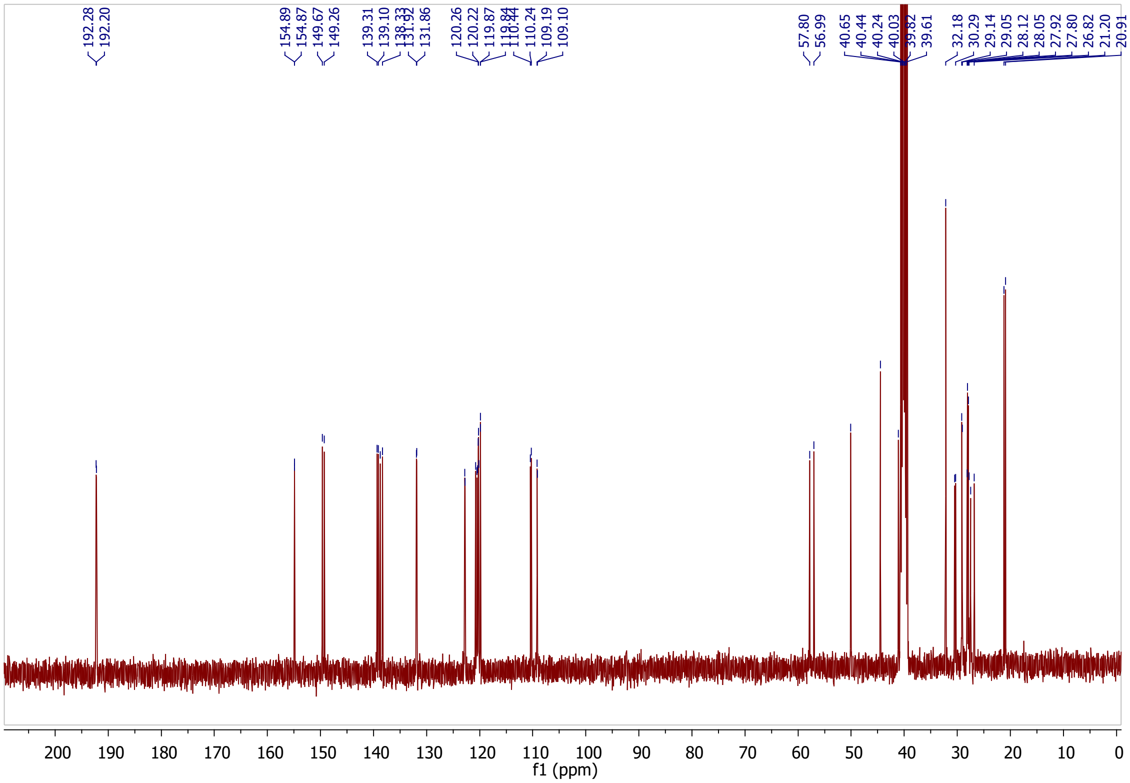

Figure S11. ^{13}C NMR spectrum of compound **3** (100 MHz).

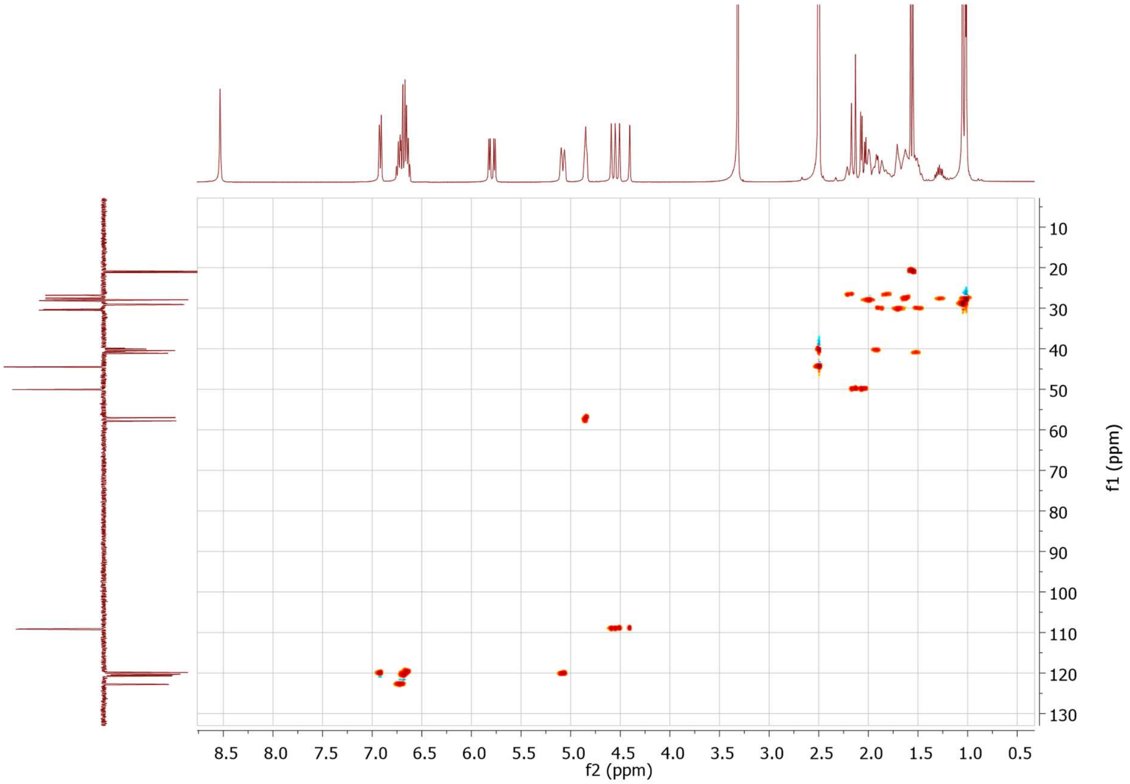

Figure S12. HSQC NMR spectrum of compound **3**.

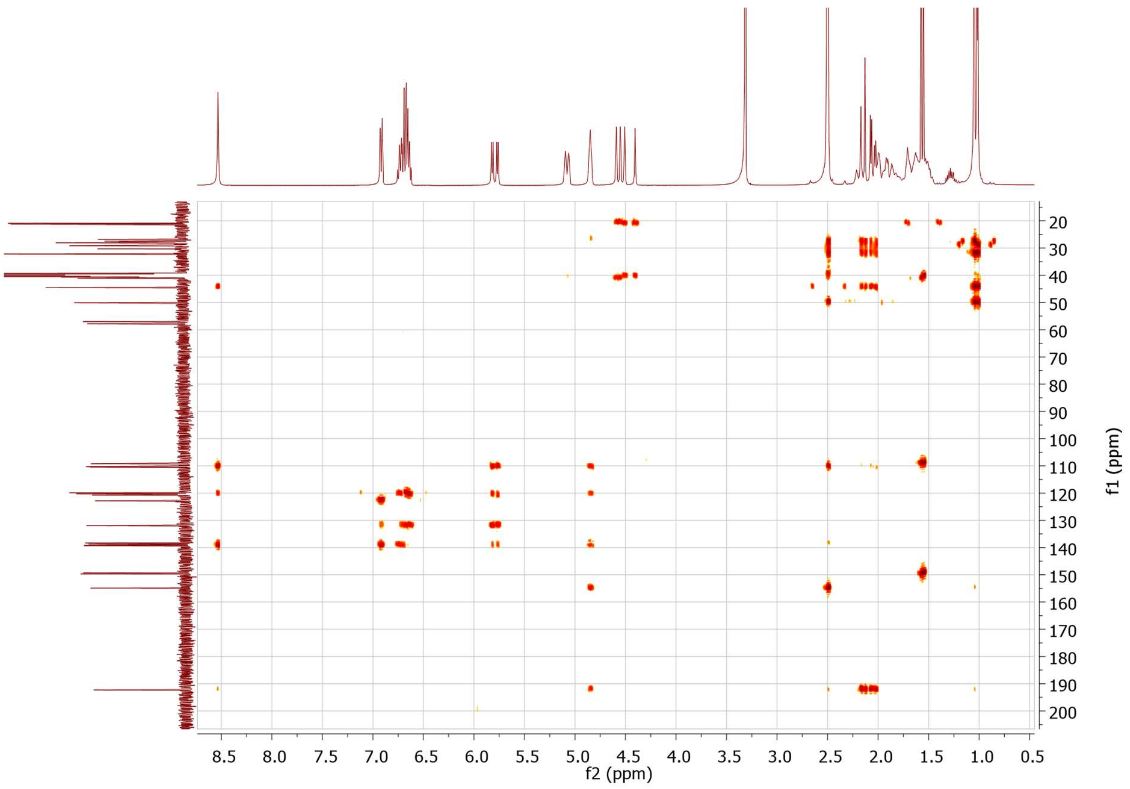

Figure S13. HMBC NMR spectrum of compound **3**.

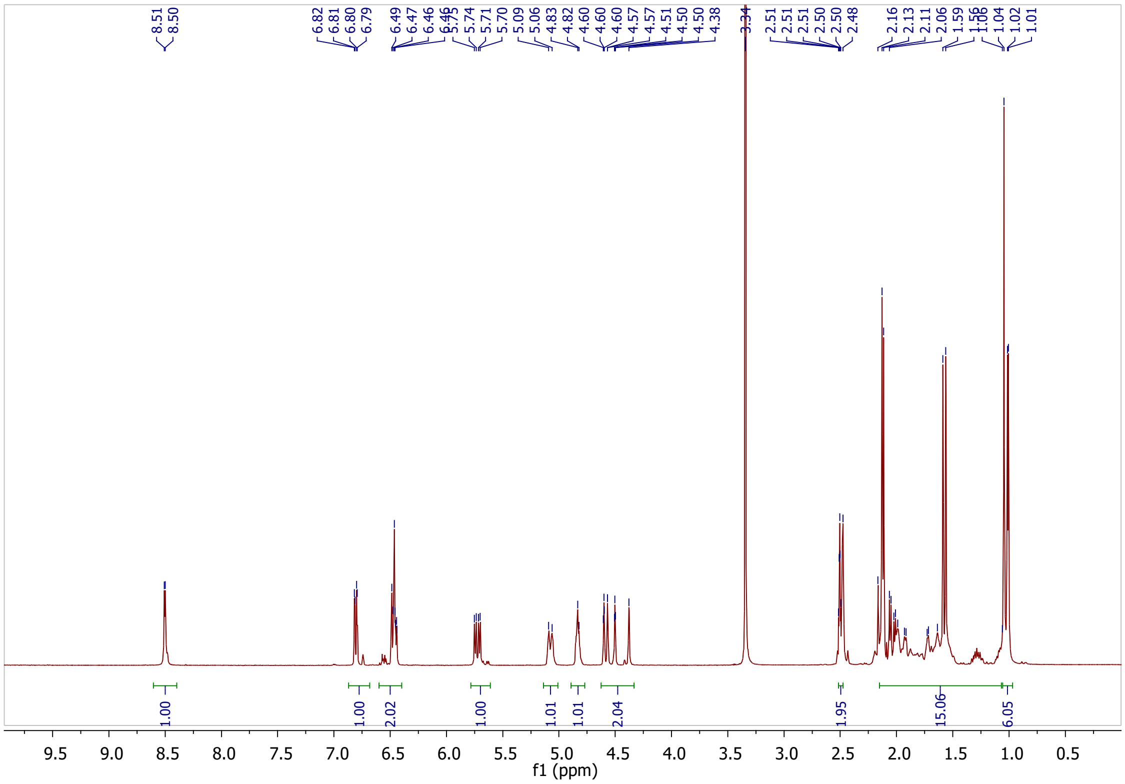

Figure S14. ^{1}H NMR spectrum of compound **4** (400 MHz).

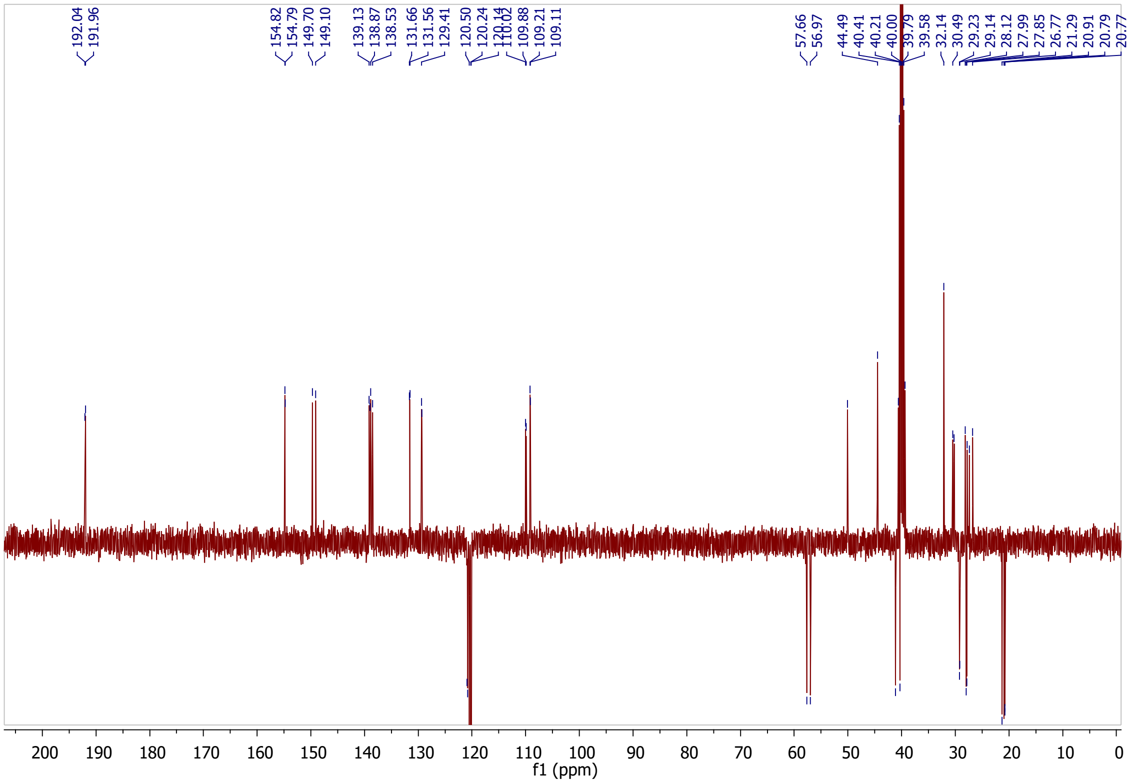

Figure S15. ^{13}C NMR spectrum of compound **4** (APT 400 MHz).

Chiral-HPLC analysis of chiral compounds and diastereomers 1-3

Conditions: Chiral-HPLC analysis of compounds **1-3** (figure S16-18) were performed on chiral stationary phase at 25°C using a CHIRALPAK® IA [Amylose-tris(3,5-dimethylphenylcarbamate) immobilized on 5 µm silica-gel, 250 x 4.6 mm ID]. The mobile phase used was hexane/acetone [isocratic mode, 50:50 (v/v)] at a flow rate of 1.0 mL/min. The UV detector was set at 220 nm (figure S21). An injection of 20 µL of 1.0 g/L concentrated samples of compounds **1-3** dissolved in the mobile phase was used. For method validation, we have analyzed racemic mixture of our previously reported benzodiazepine compounds **5** and **6** in similar chromatographic conditions (figure S19-20).

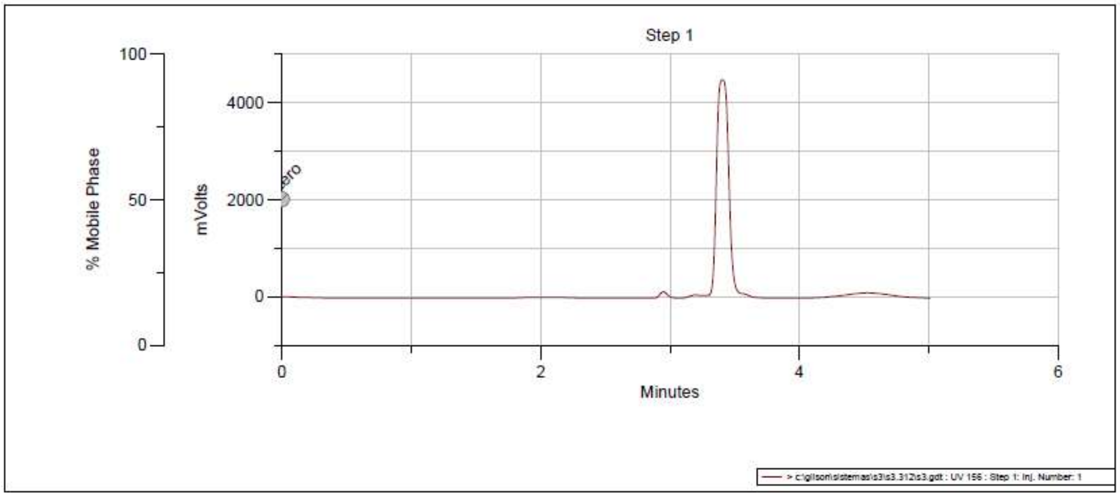

Figure S16. Chiral-HPLC analysis of compound **1**.

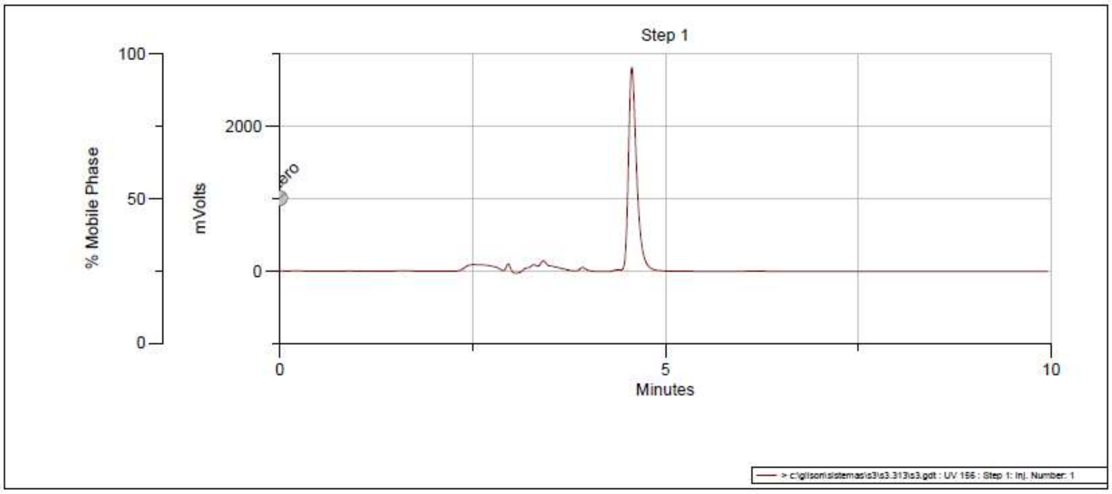

Figure S17. Chiral-HPLC analysis of compound **2**.

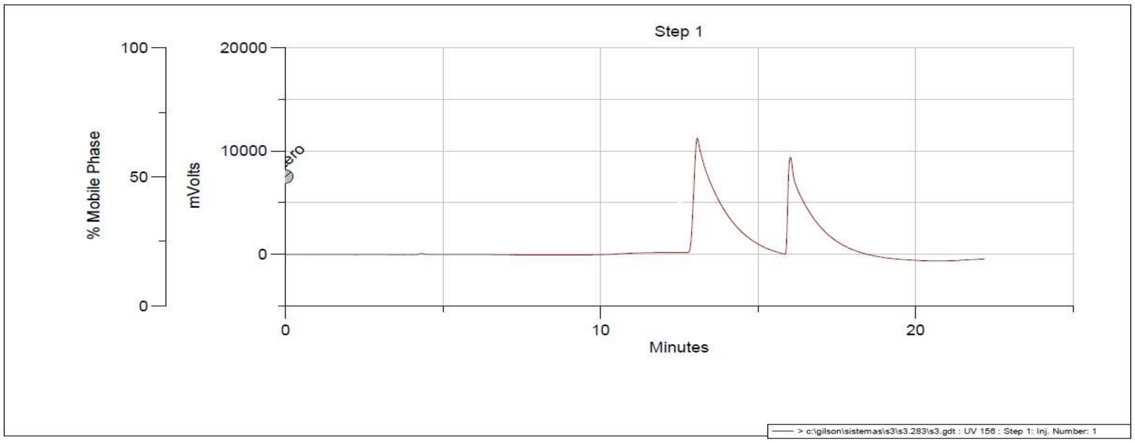

Figure S18. Chiral-HPLC analysis of compound **3**.

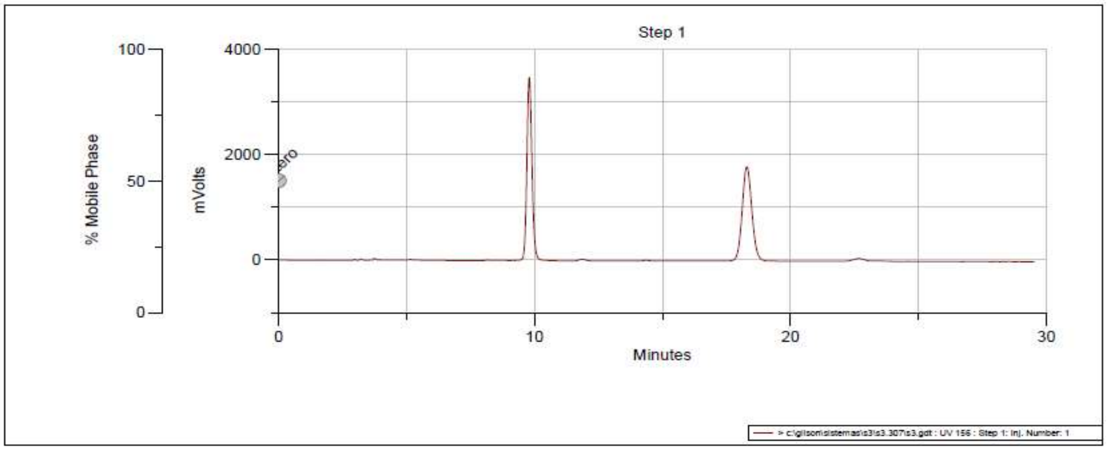

Figure S19. Chiral-HPLC analysis of the racemic compound **5** [(*E*)-8-chloro-3,3-dimethyl-11-styryl-2,3,4,5,10,11-hexahydro-1*H*-dibenzo[*b*,*e*][1,4]diazepin-1-one from: *Synlett* **2017**; 28(17): 2247-2252].

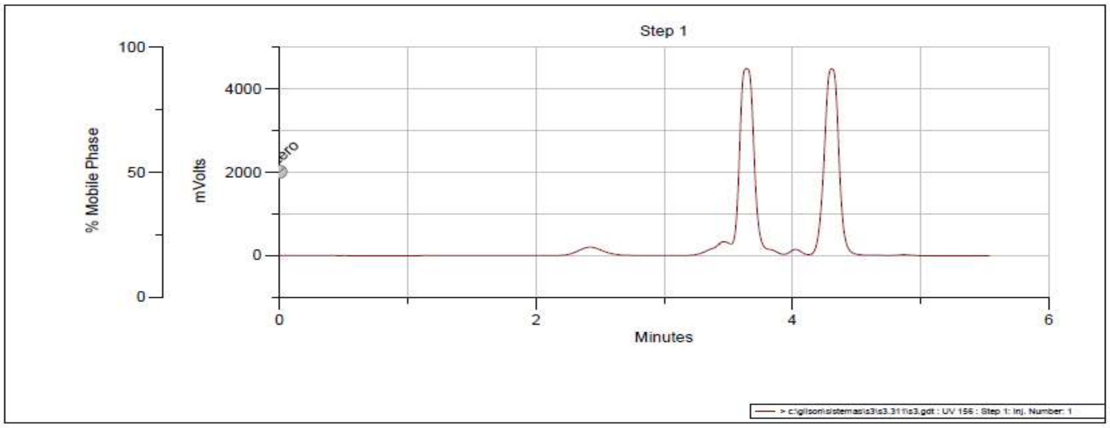

Figure S20. Chiral-HPLC analysis of the racemic compound **6** [(R/S)-1',3'-dicyclohexyl-4-(2''-hydroxyphenyl)-spiro[(*E*)-2,3-dihydro-1*H*-benzo[*b*][1,4]diazepine-2,4'-imidazolidine]-2',5'-dione from: *Synlett* **2015**; 26(02): 167-172].

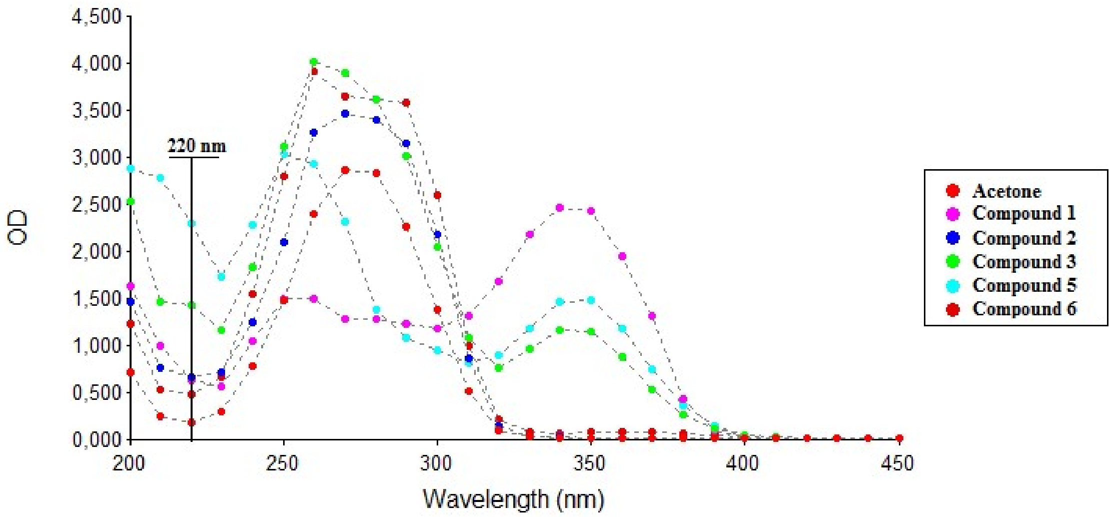

Figure S21. UV spectra showing the absorbance of compound **1-3** and **5,6** at 220 nm comparing to the absorbance of acetone.

Specific rotation analysis for pure enantiomers of compound 1 and 2:

Specific rotation is determined a KRUSS P3000 polarimeter (A. Kruss Optronic, Germany) at 589 nm and 20°C. Compounds **1** and **2** are prepared in ethanol solution according to NFT-75 113.

Compound 1	Optical rotation (degree) α^T_λ	Concentration (g/10mL)	Specific rotation $[\alpha]^T_\lambda$	Average Specific rotation
**Essay 1**	0.285	**0.02**	142.5	**+137,67** $cm^3\ dm^{-1}\ g^{-1}$
**Essay 2**	0.266	**0.02**	133.0	
**Essay 3**	0.275	**0.02**	137.5	

Compound 2	Optical rotation (degree) α^T_λ	Concentration (g/10ml)	Specific rotation $[\alpha]^T_\lambda$	Average Specific rotation
**Essay 1**	0.196	**0.02**	98.0	**+98,5** $cm^3\ dm^{-1}\ g^{-1}$
**Essay 2**	0.197	**0.02**	98.5	
**Essay 3**	0.198	**0.02**	99.0	

$$[\alpha]^T_\lambda = \alpha^T_\lambda * 100 / l * c$$

[α]: Specific rotation; l: Optical path length (1 dm); λ: Wavelength (589 nm);

T: Temperature (20°C); C: Concentration in $g/100\ cm^3$

Single-Crystal X-ray Diffraction Studies for compounds 1-4:

Single crystals of compounds **1-4** were manually harvested from the crystallization vials and immersed in highly viscous FOMBLIN Y perfluoropolyether vacuum oil (LVAC 140/13, Sigma-Aldrich) to avoid degradation caused by the evaporation of the solvent.[1] Crystals were mounted on Hampton Research CryoLoops, typically with the help of a Stemi 2000 stereomicroscope equipped with Carl Zeiss lenses.

Crystal data for compound **2** was collected at 150(2)K on a Bruker X8 Kappa APEX II CCD area-detector diffractometer (Mo Kα graphite-monochromated radiation, λ = 0.71073 Å) controlled by the APEX3 software package[2] and equipped with an Oxford Cryosystems Series 700 cryostream monitored remotely using the software interface Cryopad.[3] X-ray diffraction data for compounds **1**, **3** and **4** were, on the other hand, collected at 150(2)K on a Bruker D8 QUEST equipped with Mo Kα sealed tube (λ = 0.71073 Å), a multilayer TRIUMPH X-ray mirror, a PHOTON 100 CMOS detector, and an Oxford Instruments Cryostrem 700+ Series low temperature device. In both cases, diffraction images were processed using the software package SAINT+,[4] and data were corrected for absorption by the multiscan semi-empirical method implemented in SADABS.[5]

All structures were solved using the algorithm implemented in SHELXT-2014/5,[6] which allowed the immediate location of almost all of the heaviest atoms composing the molecular unit of the three compounds. The remaining missing and misplaced non-hydrogen atoms were located from difference Fourier maps calculated from successive full-matrix least-squares refinement cycles on F^2 using the latest SHELXL from the 2018/3 release.[7-9] All structural refinements were performed using the graphical interface ShelXle.[10]

Hydrogen atoms bound to carbon were placed at their idealized positions using appropriate *HFIX* instructions in SHELXL: *43* (aromatic carbon atoms), *13* (tertiary carbon atoms), *23* (–CH_2– carbon atoms), *137* (for the terminal methyl groups) and 123 (for the disordered methyl group). These hydrogen atoms were included in subsequent refinement cycles with isotropic thermal displacements parameters (U_{iso}) fixed at 1.2 (for the three former families of hydrogen atoms) or 1.5×U_{eq} (solely for those associated with the methyl group) of the parent carbon atoms.

For compounds **3** and **4** the hydrogen atoms associated with the N–H moieties were directly located from difference Fourier maps and included in the final structural models with the N–H distances restrained to 0.88(1) Å. The isotropic thermal displacements parameters (U_{iso}) for these hydrogen atoms were fixed at 1.5×U_{eq} of the parent nitrogen atoms.

The last difference Fourier map synthesis showed: for **1**, the highest peak (0.239 eÅ^{-3}) and the deepest hole (-0.187 eÅ^{-3}) located at 0.32 and 0.15 Å from H3F and H3C, respectively; for **2**, the highest peak (0.171 eÅ^{-3}) and the deepest hole (-0.195 eÅ^{-3}) located at 0.99 and 0.85 Å from C12 and C7, respectively; for **3**, the highest peak (0.273 eÅ^{-3}) and the deepest hole (-0.279 eÅ^{-3}) located at 0.74 and 0.56 Å from C36 and C48, respectively; for **4**, the highest peak (0.913 eÅ^{-3}) and the deepest hole (-0.424 eÅ^{-3}) located at 0.97 and 0.33 Å from C25 and H49A, respectively. Structural drawings have been created using the software package Crystal Impact Diamond.[11]

Crystal data for **1**: $C_{14}H_{16}N_4$, M = 240.31, monoclinic, space group $P2_1$, Z = 2, a = 6.1702(13) Å, b = 7.1689(15) Å, c = 15.438(3) Å, β = 99.423(4)º, V = 673.7(2)Å^3, μ(Mo-Kα) = 0.074 mm^{-1}, D_c = 1.185 g cm^{-3}, colourless plate with crystal size of 0.27×0.19×0.09 mm^3. Of a total of 4106 reflections collected, 2283 were independent (R_{int} = 0.0415). Final $R1$ = 0.0363 [$I > 2\sigma(I)$] and $wR2$ = 0.0948 (all data). Data completeness to theta = 25.24°, 99.1%. CCDC 1883076.

Crystal data for **2**: $C_{16}H_{18}N_2$, M = 238.32, orthorhombic, space group $P2_12_12_1$, Z = 8, a = 8.6559(6) Å, b = 9.8848(7) Å, c = 30.285(2) Å, V = 2591.2(3) Å^3, μ(Mo-Kα) = 0.072 mm^{-1}, D_c = 1.222 g cm^{-3}, brown block with crystal size of 0.19×0.15×0.12 mm^3. Of a total of 21777 reflections collected, 4726 were independent

(R_{int} = 0.0405). Final $R1$ = 0.0421 [$I > 2\sigma(I)$] and $wR2$ = 0.0997 (all data). Data completeness to theta = 25.24°, 99.6%. CCDC 1883077.

Crystal data for **3**: $C_{48}H_{60}N_4O_2$, M = 725.00, monoclinic, space group $I2$, Z = 4, a = 11.6743(15) Å, b = 14.0675(18) Å, c = 25.660(4) Å, β = 94.342(3)º, V = 4202.1(10) Å^3, μ(Mo-Kα) = 0.070 mm^{-1}, D_c = 1.146 g cm^{-3}, colourless plate with crystal size of 0.26×0.24×0.09 mm^3. Of a total of 19538 reflections collected, 7592 were independent (R_{int} = 0.0314). Final $R1$ = 0.0525 [$I > 2\sigma(I)$] and $wR2$ = 0.1208 (all data). Data completeness to theta = 25.24°, 99.5%. CCDC 1883078.

Crystal data for **4**: $C_{50}H_{64}N_4O_2$, M = 753.05, monoclinic, space group $I2$, Z = 4, a = 11.6925(14) Å, b = 14.2324(16) Å, c = 26.184(4) Å, β = 92.664(4)º, V = 4352.7(10) Å^3, μ(Mo-Kα) = 0.070 mm^{-1}, D_c = 1.149 g cm^{-3}, colourless block with crystal size of 0.23×0.21×0.10 mm^3. Of a total of 16198 reflections collected, 7457 were independent (R_{int} = 0.0244). Final $R1$ = 0.0650 [$I > 2\sigma(I)$] and $wR2$ = 0.1772 (all data). Data completeness to theta = 25.24°, 99.5%. CCDC 1883079.

Crystallographic data (including structure factors) for the crystal structures of compounds **1-4** have been deposited with the Cambridge Crystallographic Data Centre as the supplementary publication Nos. mentioned above. Copies of the data can be obtained free of charge on application to CCDC, 12 Union Road, Cambridge CB2 2EZ, U.K. FAX: (+44) 1223 336033. E-mail: deposit@ccdc.cam.ac.uk.

References

1. Kottke, T.; Stalke, D. *J. Appl. Crystallogr.* **1993,** 26, 615-619.
2. APEX3. *Data Collection Software Version 2016.9-0, Bruker AXS, Delft, The Netherlands* **2005-2016**.
3. Cryopad. *Remote monitoring and control, Version 1.451, Oxford Cryosystems, Oxford, United Kingdom* **2006**.
4. SAINT+. *Data Integration Engine v. 8.27b©* **1997-2012**, Bruker AXS, Madison, Wisconsin, USA.
5. Krause, L.; Herbst-Irmer, R.; Sheldrick, G. M.; Stalke, D. *J. Appl. Crystallogr.* **2015**, *48*, 3-10.
6. Sheldrick, G. M. *SHELXT-2014, Program for Crystal Structure Solution, University of Göttingen* **2014**.
7. Sheldrick, G. Crystal structure refinement with SHELXL. *Acta Crystallogr. Sect. C* **2015,** 71 (1), 3-8.
8. Sheldrick, G. M. *SHELXL Version 2014, Program for Crystal Structure Refinement, University of Göttingen* **2014**.
9. Sheldrick, G. M. *Acta Cryst. A* **2008,** 64, 112-122.
10. Hübschle, C. B.; Sheldrick, G. M.; Dittric, B. *J. Appl. Crystallogr.* **2011,** 44, 1281-1284.
11. Brandenburg, K. *DIAMOND, Version 3.2f. Crystal Impact GbR, Bonn, Germany* **1997-2010**.
